# Colorimetric-Electrochemical Combined Method for the Identification of Drugs of Abuse in Blotter Papers: A Powerful Screening Technique Using Three Analytical Responses

**DOI:** 10.1021/acsomega.5c00368

**Published:** 2025-04-18

**Authors:** Cláudia Mancilha Rocha, Larissa Magalhães de Almeida Melo, Augusto César Carvalho Santos, João Victor Coelho Pimenta, Glayton Andrade Souza, Luciano Chaves Arantes, Wellington Alves de Barros, Rodinei Augusti, Clésia Cristina Nascentes, Wallans Torres Pio dos Santos, Ângelo de Fátima

**Affiliations:** † Departamento de Química, Instituto de Ciências Exatas, Universidade Federal de Minas Gerais, Belo Horizonte, Minas Gerais 37270-690, Brazil; ‡ Departamento de Química, Universidade Federal dos Vales do Jequitinhonha e Mucuri, Diamantina, Minas Gerais 39100-000, Brazil; § Laboratório de Química e Física Forense, Instituto de Criminalística, Polícia Civil do Distrito Federal, Brasília, Distrito Federal 70610-907, Brazil; ∥ Departamento de Farmácia, Universidade Federal dos Vales do Jequitinhonha e Mucuri, Diamantina, Minas Gerais 39100-000, Brazil

## Abstract

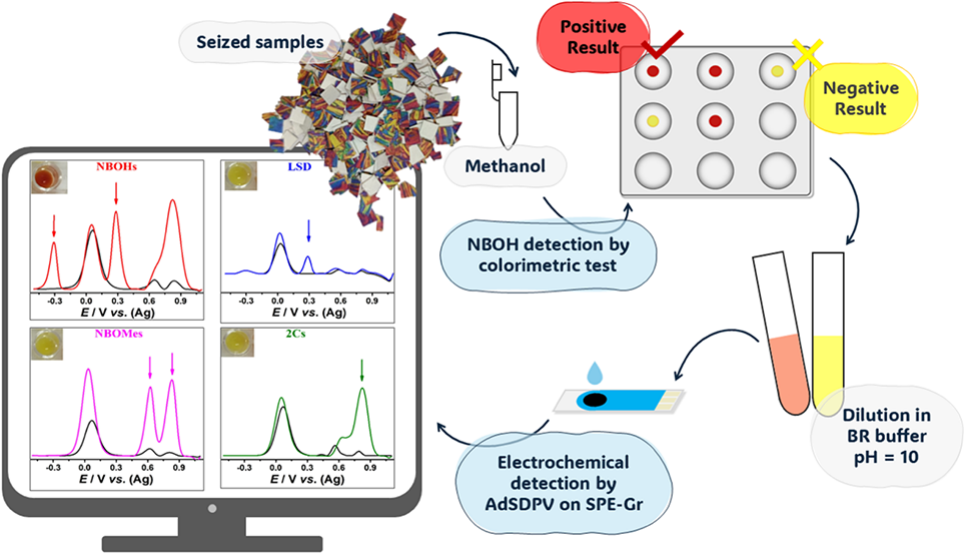

Lysergic acid diethylamide (LSD) and phenylethylamine derivatives (NBOHs and NBOMes) are commonly found on seized blotter papers, posing public health risks. Efficient screening methods for identifying these substances are currently limited. To address this, a novel protocol combining colorimetric and electrochemical techniques was developed as a screening method for drugs of abuse in blotter papers. The method uses Emerson’s colorimetric reagent (CR) for NBOH identification combined with voltammetric detection via differential pulse stripping adsorptive voltammetry (AdSDPV) using graphite screen-printed electrodes (SPE-Gr). This approach offers, for the first time, an unambiguous identification of NBOHs through three analytical responses: (1) a color change following the addition of the CR; (2) an electrochemical signal indicating the NBOHs’ redox process; and (3) a selective electrochemical signal of the colorimetric reaction product (CR-NBOH) on SPE-Gr. It also differentiates NBOH, NBOMes, 2Cs, and LSD, enabling rapid identification of drugs commonly found in blotter papers. Compared to previous sensors, this method provides selective detection of these drugs at the same pH, offering simplicity for forensic applications. The proposed method showed strong electrochemical stability with low variability (<2.3% RSD) and a low detection limit (0.3 μg mL^–1^) over a wide linear range (10–1000 μg mL^–1^), offering a simple and fast quantitative analysis of illicit drugs in these materials. The combined method was successfully applied to 33 real seized samples, with results confirmed by definitive methods.

## 1 Introduction

The identification of illegal substances in seized samples plays a vital role in the battle against drug trafficking by law enforcement. The forensic investigation typically involves two identifying steps in seized material: preliminary or screening tests, which aim to identify all possible positive samples, and confirmatory tests to pinpoint true positives among the presumed ones.^1^ Preliminary tests, usually conducted in the field, must be fast, robust, and straightforward, as they are essential in expediting the forensic process and assisting in preparing expert reports and arrest warrants. Moreover, these tests guide forensic scientists in selecting appropriate confirmatory analytical methods, making them a fundamental part of the investigative process.^2^

Lysergic acid diethylamide (LSD) is an illicit substance historically associated with blotter papers.^2^ However, according to sources such as DrugsData and internal documents from the Polícia Civil do Distrito Federal (PCDF, Brazil), the substances most commonly identified in materials sold as ″LSD″ belong to the phenethylamine class, specifically NBOMes (2-(4-X-2,5-dimethoxyphenyl)-*N*-(2-methoxybenzyl)ethanamine) and NBOHs (2-({[2-(4-X-2,5-dimethoxyphenyl)ethyl]amino}methyl)phenol), where X represents varying substituents (such as halogens, alkyl groups etc.). The 2C-series compounds (2-(4-X-2,5-dimethoxyphenyl)ethan-1-amine) are often used as precursors in the synthesis of NBOHs and NBOMes.^3−13^ Currently, some phenethylamines remain unregulated under UNODC drug conventions and are classified as new psychoactive substances (NPS). The consumption of these drugs in blotter papers has been increasing, and it is now recognized as a major public health and safety concern in several countries.^14^ Despite ongoing concerns about the use and distribution of these substances, there are still no selective preliminary identification methods for LSD, NBOHs, NBOMes, and 2C-series compounds.^15−17^

International drug control organizations, such as the Scientific Working Group for the Analysis of Seized Drugs (SWGDRUG), recommend colorimetric methods, such as Ehrlich’s reagent, for LSD identification.^2^ However, current methods are limited to confirming only the presence or absence of LSD and cannot detect NBOHs, NBOMes, or 2C-series compounds. For these other compounds, techniques such as nuclear magnetic resonance (NMR), gas chromatography (GC), mass spectrometry (MS) and Fourier transform infrared spectroscopy (FTIR) are required.^2^ Although these methods are highly robust and provide detailed structural information, they are typically reserved for confirmatory analyses rather than preliminary screening due to their high cost and limited portability.

Even these advanced techniques present challenges in selective identification. For instance, 25X-NBOHs can be misidentified as 2C-X compounds when analyzed by gas chromatography–mass spectrometry (GC-MS) with regular-length analytical capillary columns.^18^ Considering these limitations, the development of simple, selective, and reliable methods is crucial for forensic investigations involving these substances.

To address this need, some research groups have developed methods using both traditional presumptive color tests and new colorimetric tests to identify phenethylamine drugs.^19,20^ However, despite these advancements, selective colorimetric methods for NBOHs remain unavailable, and false-positive results continue to be a concern. This issue can occur due to cross-reactivity arising from similarities in chemical structure or the presence of other substances in the sample.

In parallel, electrochemical methods have gained increasing interest for detecting illicit substances in several matrices, as studies have shown.^21−25^ Electroanalytical techniques offer significant advantages, including simplicity, portability, cost-effectiveness, and rapid analysis, with selectivity and sensitivity well-suited to forensic applications. Nevertheless, selective electrochemical detection of NBOHs, NBOMes, 2C-series compounds, and LSD remains challenging. To enhance selectivity, researchers have explored robust approaches, such as employing multiple pH levels or varied working electrodes.^26−28^

The integration of colorimetric and voltammetric methods presents a promising strategy^29^ to address the challenge of identifying phenethylamine derivatives. Furthermore, both methods, whether combined or used separately, can be used in settings with limited infrastructure, enabling immediate testing at locations where these substances are seized, apprehended, or consumed. Therefore, this integration provides great potential for rapid and selective *in situ* analysis without requiring complex equipment, while also reducing costs and waste associated with forensic procedures.

Successful applications of colorimetric-electrochemical methods have already been reported for LSD and MDMA, using Ehrlich^29^ and Simon reagents,^30^ respectively. These previous reports have shown good selectivity for application in forensic samples, as the combined method offers two distinct analytical responses: (1) a color change induced by the colorimetric reagent and (2) a decrease in the oxidation signal of the investigated drug in voltammetric analysis.

However, the integration of colorimetric and voltammetric methods can be further optimized if it becomes possible to detect the product generated by the colorimetric reaction. This would provide high selectivity in forensic analysis, as it would enable three analytical responses for the preliminary identification of illicit drugs.

An innovative aspect of this study is the use of an orthogonal detection approach, which integrates two fundamentally distinct techniques—colorimetric and electrochemical—based on different physical principles. This dual-method strategy enhances the reliability of forensic analyses, particularly for official reports, by reducing the risk of false positives and improving the confirmation of results. According to the UNODC classification, colorimetric tests fall under category C, which alone is insufficient for conclusive identification, while electrochemical techniques can be classified under category B, as they provide a higher level of selectivity based on the chemical properties of the analytes. By combining these methods, the proposed approach significantly strengthens forensic evidence, as a positive result from both techniques offers greater selectivity and confidence in drug identification. This is especially valuable in forensic contexts, where robust and defensible methodologies are essential for legal proceedings. The synergy between colorimetry and electrochemistry creates a powerful analytical tool, bridging the gap between rapid screening and confirmatory analysis, and positioning it as a compelling alternative for real-world forensic applications.

In this context, we propose a combined colorimetric-electrochemical method using Emerson’s colorimetric reagent (CR),^31^ which reacts positively with phenols (NBOHs), and differential pulse adsorption stripping voltammetry (AdSDPV) on a graphite screen-printed electrode (SPE-Gr) for the selective detection of NBOHs, NBOMes, LSD, and 2C-series compounds. The proposed method offers, for the first time, three distinct responses for the unambiguous identification of NBOHs:

(1) A visible color change upon CR addition,
(2) An electrochemical signal reflecting the NBOH redox process, and
(3) A selective electrochemical signal corresponding to the CR-NBOH reaction product on the SPE-Gr.

Additionally, this approach enables differentiation between NBOMes, 2C-series compounds, and LSD, streamlining the identification of illicit drugs commonly found in blotter papers. Notably, the selective detection of these substances at the same pH is uniquely achievable through the integration of colorimetric and electrochemical techniques. For instance, the CR-NBOH reaction product displays a distinct electrochemical response compared to LSD, which cannot be differentiated in the absence of CR due to signal overlap.

This combined method is simple, rapid, sensitive, and selective, making it highly suitable for forensic applications without requiring multiple pH conditions or additional working electrodes.

## 2 Results and Discussion

### 2.1 General Procedure Applied to Seized Samples

**Figure 1:**
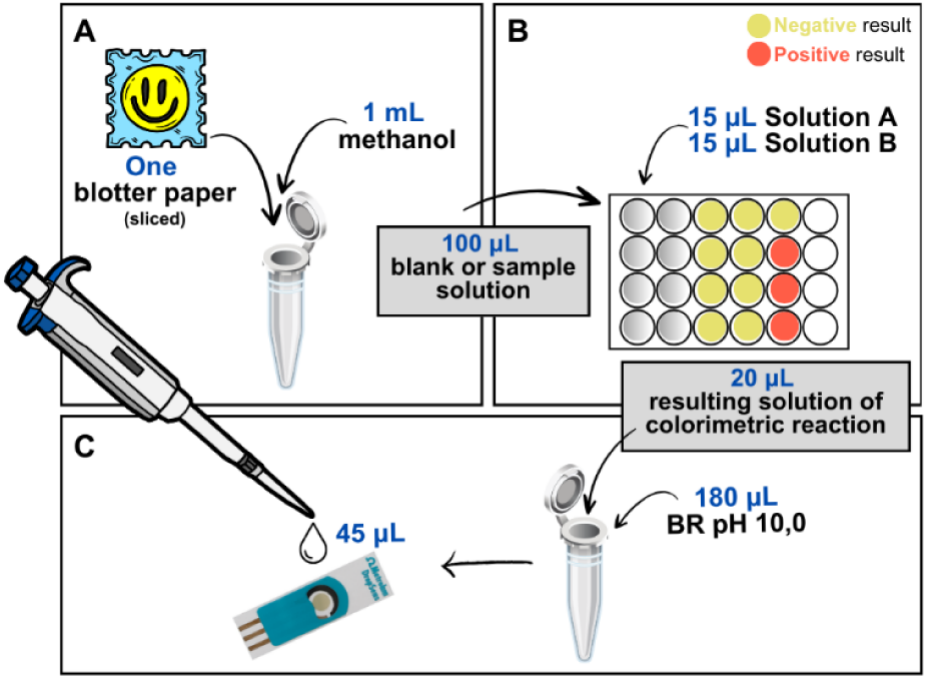
Schematic representation of the colorimetric-electrochemical test procedure: (A) methanolic extraction of the sample, followed by (B) colorimetric and (C) electrochemical analysis.

The colorimetric test was carried out in triplicate, using methanol as blank (negative standard). Each sample (blotter paper or “star”) was finely sliced or crushed and added to a microtube containing 1 mL of methanol. The microtube was vortex-agitated for 5 min, then ultrasonic irradiated for 10 min (Figure 1A). Next, 100 μL of the blank or sample solution was added to the spot plate. Then, 15 μL of solution A was sequentially added, followed by 15 μL of solution B (Figure 1B).

Subsequently, 20 μL of the resulting solution from the colorimetric test (positive or negative) was diluted in 180 μL of BR buffer at pH 10.0. After the electrochemical conditioning of the SPE-Gr electrode (as described in Section 4.5), 45 μL of the diluted solution was applied to the electrode, and the scan was performed using 1.0 min preaccumulation time, 90 mV pulse amplitude, 7.5 mV step potential, 5 ms pulse width, and 0.5 s time interval (Figure 1C).

### 2.2 Colorimetric Method

**Figure 2:**
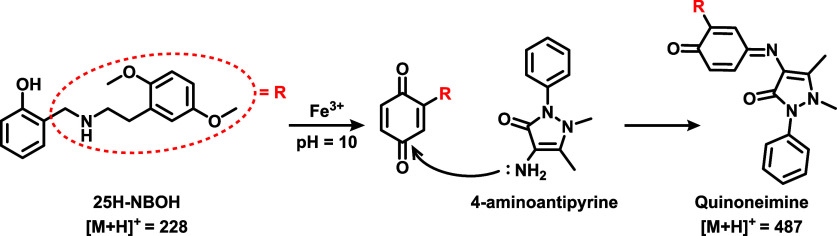
Structural representations and proton adduct masses of the reaction between 4-AAP and 25H-NBOH, yielding the chromogenic product in the presence of K_3_[Fe(CN)_6_].

In the initial step, NBOHs react with 4-AAP in the presence of an ammonium buffer solution and a potassium ferricyanide solution via Emerson’s reaction.^31^ Together, these three reagents are herein referred to as the colorimetric reagent (CR). Figure 2 shows the reaction between 25H-NBOH and 4-AAP in the presence of a ferricyanide complex, yielding a plausible product – a chromogenic compound with a mass-to-charge ratio (*m*/*z*) of 487.

At first, the chromogenic compound is likely formed through oxidation of the phenolic ring of 25H-NBOH by K_3_[Fe(CN)_6_], followed by the addition of 4-AAP, resulting in the formation of a quinoneimine. In this way, the reddish color acquired by the reaction after the addition of the colorimetric reagent (4-AAP) to the drug can be directly linked to the formation of this chromogenic compound (quinoneimine).

To confirm this hypothesis, a study employing paper spray mass spectrometry (PS-MS) was carried out. The PS(+)-MS spectrum was recorded after 5 min, as shown in Figure S1 Initially, ions at *m*/*z* 204 and *m*/*z* 288, corresponding to starting reagents (25H-NBOH and 4-AAP, respectively), were detected at low intensity. Besides, the water adduct of 25H-NBOH (*m*/*z* 306) was also observed. Note that, under reaction conditions, the ion at *m*/*z* 487 became prevalent, indicating the fast formation of an ion with the same *m*/*z* as the proposed reaction product between 25H-NBOH and the colorimetric compound 4-AAP. In addition, PS(+)-MS/MS analysis showed fragment ions at *m*/*z* 306 and *m*/*z* 308, which are consistent with the proposed structure for the ion at *m*/*z* 487 (Figure S2). Control experiments using all possible reagent combinations (25H-NBOH, 4-AAP, and K_3_[Fe(CN)_6_]) were also conducted, but none of these combinations yielded the expected product.

To assess the robustness of the proposed colorimetric method, two additional sequences were evaluated:

- **Order 1:** Blank or analyte solution + 4-AAP + ferricyanide
- **Order 2:** Blank or analyte solution + ferricyanide + 4-AAP

No significant variation in the coloration of the solutions was observed in either case (Figure S3), indicating a robust methodology. As the proposed mechanism for the colorimetric reaction, the oxidation of the phenolic hydroxyl group in the NBOHs is the key step, leading to the formation of the reactive intermediate, Order 2 (blank or analyte solution + ferricyanide +4-AAP) was chosen for subsequent experiments to maximize the production of the colored product.

The study was carried out using the compounds 25H-NBOH, 25B-NBOH, 25I-NBOH, MD-NBOH, and 34-NBOH as analytes to evaluate the potential influence of substituents on the colorimetric reaction and the resulting color changes. Unlike the others, the MD-NBOH compound produced a more orange hue but still yielded results that were easily distinguishable from the blank (Figure S4). Additionally, compounds 25H-NBOMe, and 2C–H were also evaluated, and both showed negative results in the colorimetric test.

### 2.3 Electrochemical Method

#### 2.3.1 Electrochemical Behavior of 25H-NBOH and Its Product in the Colorimetric Reaction

25H-NBOH was chosen as the model molecule and assessed in a 0.1 mol L^–1^ BR buffer in the pH range of 2.0 to 12.0, in the absence (A) and presence (B) of CR, on SPE-Gr by cyclic voltammetry (CV), as depicted in Figure S5. The electrochemical behavior of 25H-NBOH on SPE-Gr shows five irreversible oxidation processes (O_1_, O_2_, O_4_, O_5_, and O_6_) and a reversible redox pair (O_3_/R_1_). Processes O_1_, O_2_, and O_5_ appear only in the second scan (Figure S6), while process O_6_ is observed only from pH 7.0 forward (Figure S5A). An external reference electrode (Ag/AgCl) was used to ensure the accuracy of the peak potential (*E*_*p*_) dependence on pH, and a distinct SPE was used for each pH.

A pH-dependent behavior of the redox processes of 25H-NBOH on SPE-Gr was also observed since the peak potentials (*E*_*p*_) of these electrochemical processes shifted to more negative potentials with increasing pH. Figure S7A shows plots of *Ep* vs pH for 25H-NBOH redox processes on SPE-Gr and seven linear ranges were observed (namely (1S)–(7S)), shown in Table S1. The slopes obtained for O_3_/R_1_ are very close to the theoretical value of 0.0592 V/pH, indicating that an equal number of electrons and protons are involved in this 25H-NBOH reversible redox process. Furthermore, at pHs above 7.0, there is a better correlation with peak current, providing an adequate response in the detection of this compound (Figure S7B). This can be observed for the O_1_ and O_2_ 25H-NBOH processes, both in the absence and presence of CR (Figure S8). BR buffer at pH 10 was selected as the supporting electrolyte for the detection of 25H-NBOH because an oxidation process (P_oxi_*) was observed only at this pH in the presence of CR (Figure 2B). This process occurs exclusively when CR reacts specifically with NBOHs, suggesting that P_oxi_* corresponds to the oxidation of the product formed in the colorimetric reaction between NBOHs and CR. Since this oxidation process (P_oxi_*) enhances the selectivity of the method, pH 10 was chosen for all subsequent experiments.

The mass transport control of 25H-NBOH redox reactions, both in the absence and presence of CR on the SPE-Gr surface, was evaluated using CV at different scan rates (*v*) in a 0.1 mol L^–1^ BR buffer solution at pH 10.0 (Figures S9A and S10A, respectively). The anodic peak currents (*I*_*pa*_) for O_1_, O_2_, O_6_, and P_oxi_* were more proportional to the scan rate (*R*^2^ = 0.99 for O_1_, O_2_ and P_oxi_*, *R*^2^ = 0.984 for O_6_; Figures S6B and S7B) and were also proportional to the square root of scan rate (*R*^2^ = 0.99 for P_oxi_* and O_6_, *R*^2^ = 0.95 for O_1_ and 0.98 for O_2_, Figures S9C and S10C), indicating partial adsorption-controlled electrochemical processes on the SPE-Gr surface. This was further confirmed by the logarithmic plot of *I_pa_* vs *v*, which showed linear relationships (namely (8S)–(11S) - Table S2) with slope values of 0.85, 0.76, 0.53, and 0.64 for O_1_, O_2_, O_6_, and P_oxi_*, respectively, indicating that these electrochemical processes are controlled by both diffusion and adsorption mechanisms.^32^

#### 2.3.2 Determination of 25H-NBOH in the Presence of CR by AdSDPV

Initially, the electrochemical response of 25H-NBOH was investigated using differential pulse voltammetry (DPV) and square wave voltammetry (SWV) techniques. A more pronounced voltammetric signature and enhanced peak currents were observed with the DPV method. Given this and the observation of adsorption phenomena in the electrochemical behavior of 25H-NBOH and CR on SPE-Gr, the AdSDPV method was chosen to improve the sensitivity and selectivity of the voltammetric approach.

**Figure 3:**
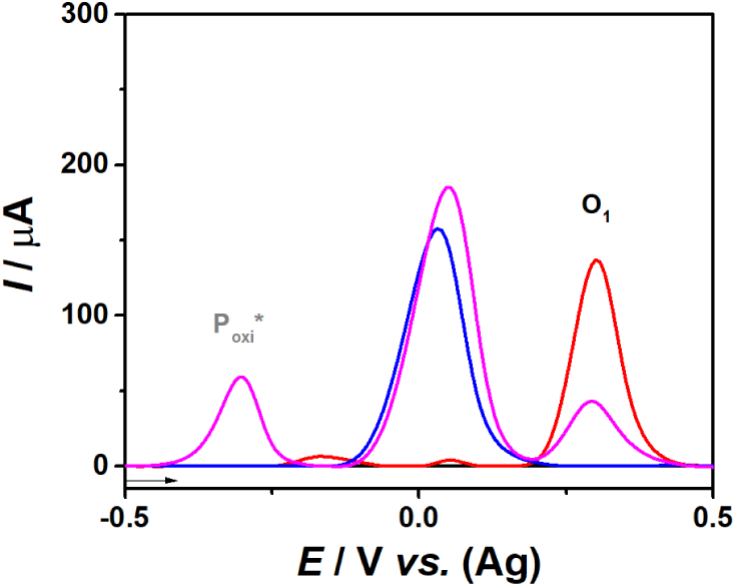
AdSDPV voltammograms in 0.1 M BR buffer solution pH 10.0 on SPE-Gr before (black line) and after the addition of 0.4 × 10^–3^ mol L^–1^ 25H-NBOH (red line), CRs (blue line), and 25H-NBOH + CRs (magenta line).

The AdSDPV parameters were systematically optimized to maximize peak current intensity and peak width at half-height, with optimal conditions identified at an amplitude of 90 mV, a step potential of 7.5 mV, a modulation time of 50 ms, and an interval time of 0.5 s. The preaccumulation duration on the SPE-Gr substrate was also examined, selecting 1.0 min as the optimal condition to sustain a rapid forensic analysis while still achieving a noticeable enhancement in peak current. The electrochemical behavior of CR, 25H-NBOH, and their mixture under these optimized conditions is depicted in Figure 3.

Figure 3 illustrates that the voltammogram of the isolated colorimetric reagents (blue line) presents a pronounced signal at +0.031 V, attributed to the presence of the K_3_[Fe(CN)_6_] redox probe. On the other hand, isolated 25H-NBOH (red line) presents an oxidative process with a signal at +0.299 V (O_1_) vs. Ag, which is characteristic of the NBOHs group and independent of the colorimetric reaction.

Notably, the +0.299 V signal does not overlap with any other redox process of the isolated colorimetric reagents (blue line), making it a reliable indicator of the presence of this phenylethylamine group. Furthermore, Figure 3 also reveals that after the colorimetric reaction between CR and NBOHs, a distinct oxidation process (P_oxi_*) is observed at −0.3 V vs. Ag. This process is exclusively observed in a positive colorimetric reaction, confirming that it corresponds to the oxidation of a product generated from the interaction between NBOHs and CR. This provides an additional unique analytical marker for the detection of NBOHs using the proposed method.

Using the method proposed here, the initial indication of the presence of NBOHs in the sample is given by the color change (yellow to red) in the Emerson test. In the electrochemical stage, this is confirmed through the appearance of the oxidation process characteristic of NBOHs (O_1_), as well as the appearance of the oxidation signal of the reaction product between NBOHs and CR (P_oxi_*). The results highlight the potential of the proposed method as a viable alternative for preparing a robust, reliable, and rapid expert report for the direct selective identification of NBOHs.

**Figure 4:**
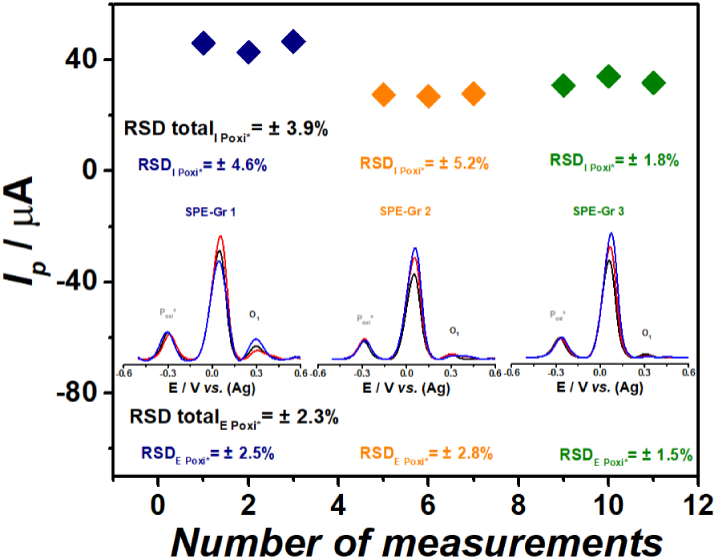
*I*_p_ vs. number of measurements on three different SPE-Grs, inserted AdSDPV voltammograms of 0.4 × 10^–3^ mol L^–1^ 25H-NBOH + CRs in 0.1 mol L^–1^ BR buffer solution pH 10.0. Inserted data on RSD for *E*_*p*_ and *I*_*p*_ of P_oxi_^*^.

In this experimental setup, assessments of repeatability and reproducibility were conducted utilizing both the same (*N* = 3) and varied (*N* = 3) SPEs-Gr electrodes, with a concentration of 0.4 × 10^–3^ mol L^–1^ 25H-NBOH and the presence of CR, as depicted in Figure 4.

As shown in Figure 4, the analysis of 25H-NBOH and CR solutions via AdSDPV, using both the same or different electrodes, yielded low relative standard deviations (RSDs), with an overall RSD for *I*_*p*_ of 3.9% (P_oxi_*) and 4.2% (O_1_). This underlines the reliability and reproducibility of the developed method.

Furthermore, the anodic peak potential (*E*_*pa*_) of O_1_ and P_oxi_* were consistent across all measurements, with a total RSD of 2.3% for both O_1_ and P_oxi_*, even when employing pseudo-RE electrodes. These findings suggest that the combination of SPE-Gr with the AdSDPV technique offers a viable screening technique for the detection of NBOHs, particularly when CR is utilized. Importantly, the proposed method uses the *E*_*pa*_ values of O_1_ and P_oxi_* for the initial selective detection of NBOHs-group in seized samples.

**Figure 5:**
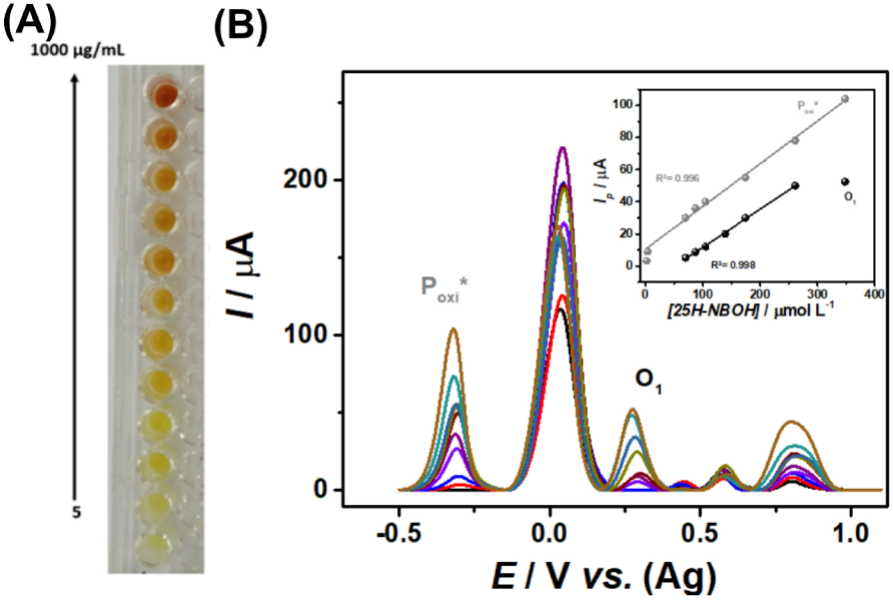
(A) Results of the Emerson test for 25H-NBOH solutions in concentrations from 0.1 to 40 × 10^–3^ mol L^–1^ and (B) AdSDPV voltammograms of these solutions diluted 10× in 0.1 mol L^–1^ BR pH 10. [25H-NBOH]: 1.74, 3.48; 8.7; 17.4; 34.8; 69.6; 87.0; 104.4; 139.2; 174.0; 261.0; 348.0 μmol L^–1^; inserted linear regressions *Ip* vs. [25H-NBOH].

The determination of the linear working range for quantifying NBOHs through the proposed method was assessed using solutions with concentrations spanning from 5 to 1000 μg mL^–1^ (0.1–40 × 10^–3^ mol L^–**1**^) of the model molecule 25H-NBOH for the colorimetric assay (Figure 5A). Subsamples from these solutions were subsequently diluted 10-fold in BR buffer solution at pH 10.0 for electrochemical detection (Figure 5B).

As can be seen in Figure 5A, while the lowest detectable concentration by the colorimetric technique was 100 μg mL^–1^ (87 μmol L^–1^), the lowest value detectable by the electrochemical technique was 5 μg mL^–1^ (1.74 μmol L^–1^), showing in Figure 5B. Moreover, the lowest quantifiable concentration by the electrochemical technique was 10 μg mL^–1^ (3.48 μmol L^–1^). The colorimetric solutions were diluted and analyzed by the electrochemical method, which showed linearity between 10 and 1000 μg mL^–1^ (3.48–348 μmol L^–1^), monitoring P_oxi_*, with regression eq 1 and *R*^2^ of 0.996:
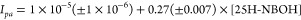
1

As can be seen in Figure 5B, it is also possible to quantify NBOHs by the electrochemical signal of O_1_, whose linear regression obtained for this 25H-NBOH process is given by eq 2, with *R*^2^ = 0.998:
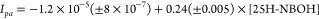
2

The theoretical limits of detection and quantification (LOD and LOQ) for the model molecule, 25H-NBOH, were calculated to be 0.3 μg mL^–1^ and 10 μg mL^–1^ (0.9 and 3.1 μmol L^–1^), obtained from *3S*_*b*_/*m* and *10S*_*b*_/*m*, respectively, where *S*_*b*_ is the standard derivation (*N* = 10) of the background response and *m* is the slope of the linear regression equation.

The detected concentration levels for NBOHs are sufficiently low for application in real samples utilizing the colorimetric-electrochemical proposed method. Thus, the developed method is highly sensitive for screening NBOHs in forensic samples, employing the combination of colorimetric and electrochemical tests with quantification potential for this class. Furthermore, calibration curves for other compound classes and additional NBOH compounds were obtained and are presented in Figure S11, with the corresponding linear regression equations (namely (12S)–(15S)) described in Table S3.

#### 2.3.3 Differentiation between NBOHs, NBOMes, LSD, and 2Cs in the Presence of CR by AdSDPV

**Figure 6:**
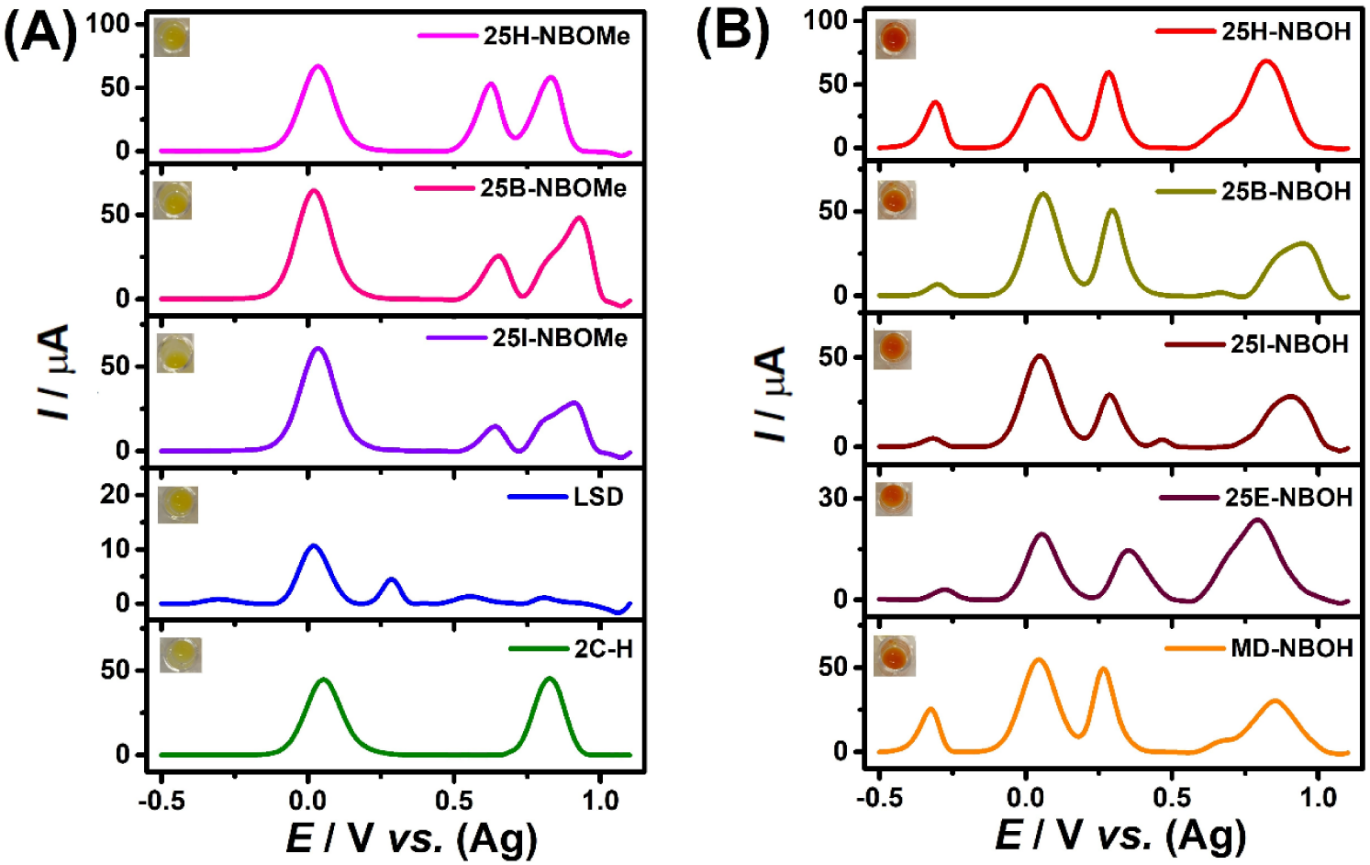
AdSDPV voltammograms on SPE-Gr for NBOMes, LSD and 2C–H (A) and NBOHs (B). All compounds were at a concentration of 0.4 × 10^–3^ mol L^–1^ in 0.1 mol L^–1^ BR buffer solution pH 10.0 (in the presence of the CRs). Emerson test results are inserted.

The method’s applicability to other compounds within the same class (25B-NBOH, 25I-NBOH, and MD-NBOH) and other classes: NBOMes (25H-NBOMe, 25B-NBOMe, and 25I-NBOMe), 2C–H, and LSD is demonstrated in Figure 6. Previously, obtaining easily distinguishable electrochemical profiles for each of these compound classes using electrochemical tests was only possible using at least two different pHs in the analysis, or more than one type of electrode, being a very laborious procedure for field application.^33^

**Scheme 1:**
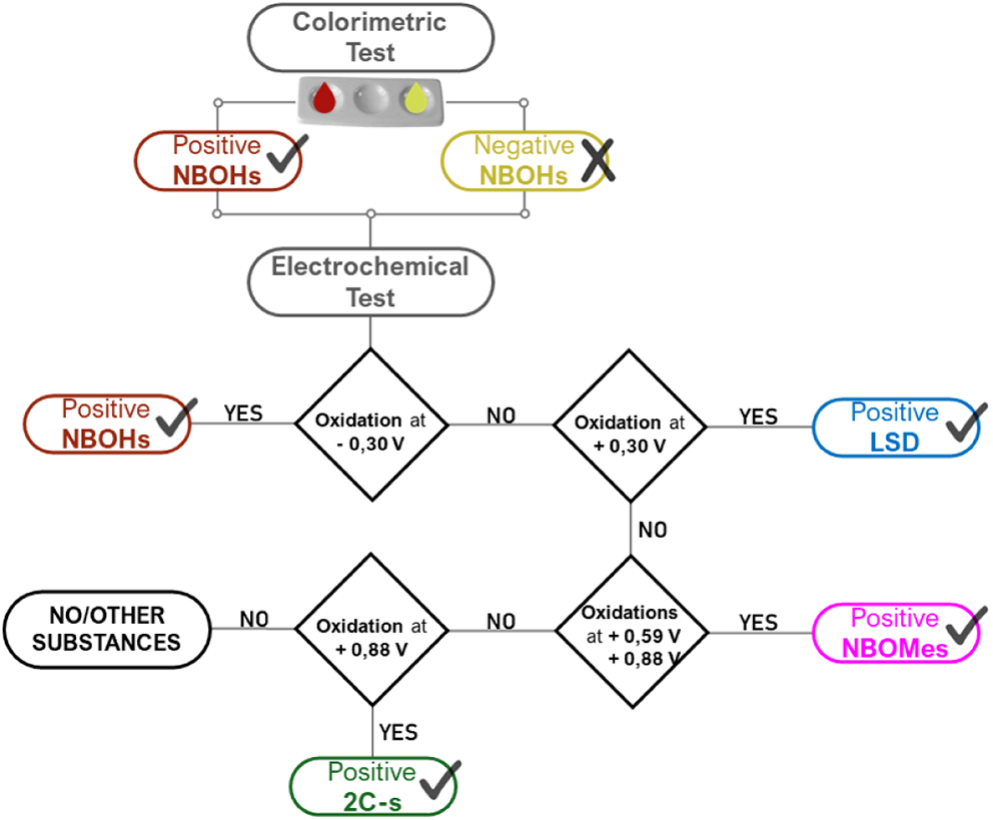
Interpretation Diagram of the Results Obtained by the Proposed Method

As can be seen in Figure 6, all NBOHs analyzed by the proposed method presented P_oxi_* oxidation products, indicating that a chemical reaction occurs between CR and the NBOHs group of drugs. Highlighting that the colorimetric test was selective for all NBOH class (Figure 6B). In contrast, as predicted, compounds from the NBOMes, 2Cs, and LSD categories did not exhibit any colorimetric reaction. However, these compounds showed characteristic electrochemical behavior, whose redox processes are sufficiently resolved and distinct from those obtained for NBOHs, which allow a differentiation between these four classes (Scheme 1).

The combined method proposed herein innovates in the detection and differentiation of NBOHs, LSD, NBOMes, and 2Cs groups. This differentiation can be achieved following the colorimetric reaction, by evaluating both the absence of a color change and the presence of the oxidation signal of P_oxi_*. Under these conditions, the absence of drugs from the NBOHs group in forensic samples can be confidently confirmed. Consequently, a subsequent assessment of redox processes using SPE-Gr is warranted to ascertain the presence of other psychoactive substances. This is facilitated by the previously established and characteristic *E*_*p*_ values for these substances, as illustrated in Figure 6:
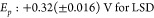

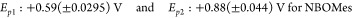

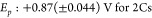


It is important to highlight that without the colorimetric test, the peak potential of NBOHs (O_1_ = +0.30 V) overlaps the peak potential of LSD (+0.312 V), making it impossible to differentiate between the two compounds.

In summary, the results obtained through the proposed colorimetric-electrochemical method are succinctly interpreted and presented in Scheme 1.

### 2.4 Determination of NBOHs, NBOMes, 2Cs, and LSD in Real Seized Samples

**Figure 7:**
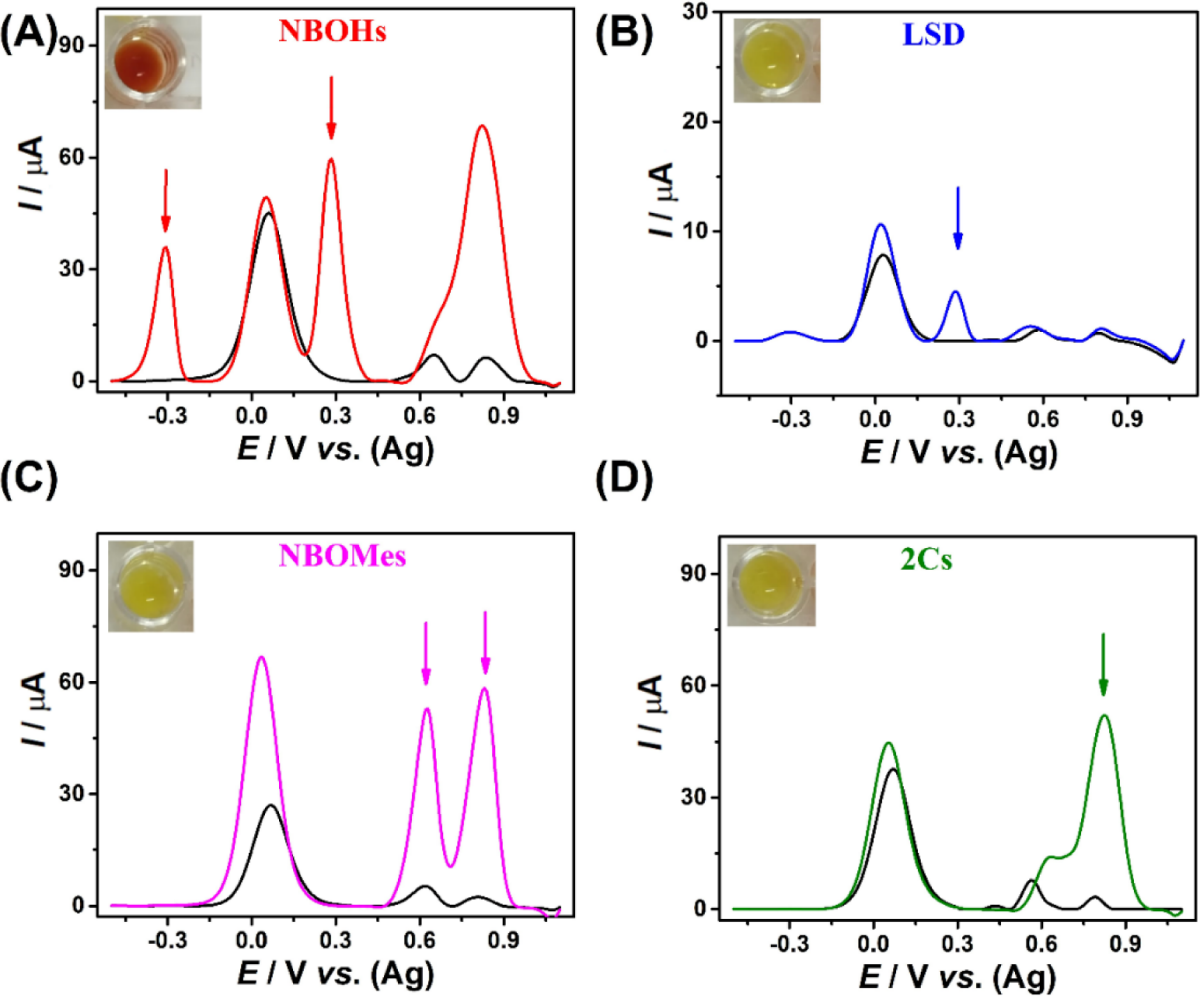
AdSDPV voltammograms recorded in 0.1 mol L^–1^ BR buffer solution pH 10.0 on SPE-Gr for seized samples containing 25B-NBOH (red) (A), LSD (blue) (B), 25B-NBOMe (magenta) (C), and 2C–B (olive) (D).

Finally, a blind test of this method was performed on 33 real blotter samples seized by the Polícia Civil do Distrito Federal in Brazil (PCDF), yielding consistent results with prior analyses by PCDF. These analyses were conducted using an innovative gas chromatography–mass spectrometry method developed by PCDF.^18^ The appearance of some of the seized blotter papers, as well as the results obtained by the proposed method (screening) and the definitive method are shown in Table S4. Additionally, four examples of these seized samples are shown in Figure 7.

Remarkably, all samples containing NBOHs drug were correctly identified. Additionally, the method reliably detected LSD, NBOMes, and 2Cs with 100% accuracy (Table S4), showcasing its applicability and high selectivity for detecting NBOHs, NBOMes, LSD, and 2Cs in confiscated blotter paper samples.

The reliability, accuracy, and reproducibility of the proposed method were validated through confirmatory tests conducted by the Polícia Civil do Distrito Federal (PCDF) on the 33 analyzed samples. A selection of these results is presented in Figure S12. Furthermore, addition-recovery studies performed on three samples are detailed in Figure S13 and Table 1.

**Table 1:** Addition-Recovery Studies for NBOH, NBOMe, and LSD Classes

Drug Class	Concentration add (μmol L^–1^)	Recovery ± RSD (%)
NBOH	85.00	90.6 (±9.0)
NBOMe	85.00	104.6 (±6.2)
LSD	85.00	110.8 (±3.3)

As shown in Table 1, the proposed method exhibits high accuracy and no significant matrix effect, with recovery values close to 100% for NBOHs, NBOMes, and LSD. However, the study did not include the 2C class due to the limited availability of reference standards for these compounds.

To the best of our knowledge, no single colorimetric or electrochemical method can identify all four drug classes examined in this study. Electrochemical methods typically require multiple pH conditions or different working electrodes, while existing colorimetric methods often lack sufficient selectivity. For comparison, Table 2 summarizes the key analytical parameters of previously published methods for the detection of NBOHs, NBOMes, 2Cs, and LSD.

**Table 2:** Comparison of Analytical Parameters

Method	Drug Class	Linear range	LOD	Ref.
Electrochemical—SWVs	LSD	0.16–40 mmol L^–1^	0.05 mmol L^–1^	(34)
Colorimetric	NBOMes	-	225 μg	(35)
Electrochemical—SWVs	LSD	4.8–101.6 μmol L^–1^	0.45 μmol L^–1^	(27)
Electrochemical—SWVs	LSD, NBOHs, and NBOMes	20–100 and 20–70 μmol L^–1^	0.01 and 1.13 μmol L^–1^	(26)
Electrochemical + Colorimetric	LSD, NBOHs, NBOMes, and 2Cs	3.48–348 μmol L^–1^	0.9 μmol L^–1^	This work

Table 2 demonstrates that the detection of LSD was achieved without interference from NBOHs, NBOMes, and 2Cs [26]. However, the method was neither optimized nor evaluated for the simultaneous detection of all four drug groups. Specifically, the reliability of the electrochemical response, calibration curves, and method accuracy were assessed solely for the detection of LSD. Similarly, other authors have demonstrated the detection of only three out of the four mentioned classes. Moreover, achieving selective detection of LSD, NBOHs, NBOMes, and 2Cs required the use of multiple working electrodes or varying pH conditions,^26,27^ making the proposed electrochemical methods more complex compared to the combined colorimetric-electrochemical approach presented in this study. Additionally, while the colorimetric detection of NBOMes has been demonstrated, it does not extend to the other drug classes.^35^ Therefore, the proposed combined colorimetric-electrochemical method introduces, for the first time, the ability to selectively detect four of the most commonly found drug classes in blotter papers in a simple, rapid, sensitive, and highly selective manner.

## 3 Conclusions

This study presents a combined colorimetric-electrochemical method for the rapid and straightforward preliminary identification of four classes of illicit drugs—NBOHs, NBOMes, 2Cs, and LSD—in seized blotter papers. Notably, the proposed method achieves, for the first time, the unambiguous identification of NBOHs through three distinct analytical responses. By integrating a colorimetric test with electrochemical detection on screen-printed graphene electrodes (SPE-Gr) using adsorptive stripping differential pulse voltammetry (AdSDPV), this approach enables sensitive and reproducible detection and quantification of NBOHs, NBOMes, 2Cs, and LSD in seized samples. The method also demonstrates excellent stability (RSD < 2.3%) for drug identification based on peak potentials (*N* = 3) using the same or different SPE-Gr electrodes. The combined method requires a minimal sample volume (45 μL), making it portable and suitable for on-site analysis. It achieves a low detection limit (0.3 μg mL^–1^) and a wide linear range (10–1000 μg mL^–1^) through electrochemical detection, enabling simple and rapid quantitative analysis of illicit drugs in seized samples. This work introduces an innovative methodology to address the challenges of identifying and distinguishing phenylethylamines and LSD in blotter paper samples. It provides forensic analysts with a reliable, selective tool that enhances identification capabilities while supporting efficient and cost-effective field applications.

## 4 Methods

### 4.1 Chemicals and Samples

All solutions were prepared with deionized water with a resistivity of not less than 18.2 MΩ.cm^–1^ (at 25 °C) obtained using the Milli-Q system (Millipore, USA). The reagents 4-aminoantipyrine (ACS Científica), potassium ferricyanide (Neon), NH_4_OH concentrated solution (Sigma-Aldrich), and NH_4_Cl (Synth) were used to colorimetric test. The analytical standard of 25H-NBOH, 25B-NBOH, 25I-NBOH, MD-NBOH, 34-NBOH, 25H-NBOMe, 25B-NBOMe, 25I-NBOMe and 2C–H, were obtained by the synthesis using the general procedure and characterized according to the methodology already published by the research group,^36^ as described in Supporting Information, and solubilized in HPLC-grade methanol to obtain stock solutions (1.0 × 10^–2^ mol L^–1^). 25E-NBOH and LSD analytical standards were provided by the United Nations Office on Drugs and Crime (UNODC) and solubilized in HPLC-grade methanol, purchased from JT Baker Chemicals. These solutions were diluted in a supporting electrolyte for electrochemical measurements. Britton–Robinson (BR) buffer solution (0.1 mol L^–1^) at pH 10.0 was used as a supporting electrolyte.

Thirty-three real samples seized (stamps and “stars”) were investigated by the proposed method in a blind test for the application of preliminary identification tests. All samples were obtained following an extraction step performed in the Polícia Civil do Distrito Federal in Brazil (PCDF), Brazil.

### 4.2 Instrumental and Apparatus

To confirm the reaction mechanism, paper spray mass spectrometry (PS-MS) experiments were conducted on a Thermo LCQ-Fleet mass spectrometer (ThermoScientific, San Jose, USA) in the positive ion mode. The instrumental conditions were as follows: voltage applied to the paper, 5 kV; capillary temperature, 275 °C; capillary voltage, 8 V; tube lens voltage, 70 V. Full scan mass spectra were acquired over a 50–500 *m*/*z* range. The triangular paper’s tip was placed in front of the mass spectrometer inlet at approximately 5 mm.

All voltammetric experiments were carried out using a μPGSTAT 101 N potentiostat (Metrohm Autolab BV, Utrecht, Netherlands) controlled by the NOVA 2.1 software, after bubbling (5 min) pure nitrogen gas was in the studied solutions, to remove oxygen. The electrochemical behavior of all compounds was studied using commercial SPEs from Metrohm DropSens (Oviedo, Spain), with a 4 mm diameter graphite working electrode (SPE-Gr, model DRP-110), a carbon auxiliary electrode, and a silver pseudoreference electrode.

Seized samples (33) were also analyzed using a GC-MS model 7890A/5975C (Agilent Technologies, Santa Clara, CA, USA). Agilent MSD ChemStation and Enhanced ChemStation Data Analysis software [version E.02.02.1431] were used for data acquisition and analysis. Mass spectral deconvolution, retention time collection, and identification of detected analytes were performed using the Automated Mass Spectral Deconvolution and Identification System (AMDIS) [version 2.73 Build 149.31] and the National Institute of Standards and Technology Mass Spectral Search Program (NIST Search) [version 2.3]. The AMDIS parameters were set to the *component* mode, the *simple* GC-MS data analysis type, and the *default* analysis settings. Furthermore, the analytes were identified using commercially available spectral libraries, such as SWGDRUG [version 3.11] and the Cayman Spectral Library [version 14092022].

The GC-MS consumables used in the analysis were as follows: an ultrainert liner (Agilent, part number 5190-2294), a J&W DB-1 ms ultrainert analytical column (4 m × 0.25 mm ID × 0.25 μm film thickness; Agilent, part number 122-0132UI), and 4 mL/min of ultrapure helium as the carrier gas. An injection volume of 1 μL and a 20:1 split ratio was used. Inlet temperature was set to 250 °C, and the oven temperature program started at 50 °C, followed by a ramp of 20 °C/min until a final temperature of 280 °C was reached, and this was maintained for 5 min (the total run time was 16.5 min). A solvent delay of 1.0 min was applied, the mass spectrometer was set at full-scan mode acquisition (40–550 *m*/*z*) with an ionization energy of 70 eV, and the transfer line, ion source, and quadrupole temperatures were set at 280, 300, and 150 °C, respectively.

### 4.3 Colorimetric Procedure

The model molecule used in the studies was 25H-NBOH at a concentration of 1.0 mg mL^–1^, and the colorimetric solutions were prepared as described in Supporting Information. The order of addition of the colorimetric reagents (4-AAP and K_3_[Fe(CN)_6_]) was varied to assess the robustness of the methodology. The influence of common interferents (such as 2C–H, and 25H-NBOMe) was evaluated, along with the reproducibility of results using different analytes from the same class of compounds (25B-, 25I-, MD-, and 34H-NBOH). During the optimization process of the colorimetric test, images were recorded using a Motorola G7 Play cell phone.

### 4.4 Mass Spectrometry Experiments

On-surface reaction test was conducted by dropping to triangular paper (1 × 1.5 × 1.5 cm), 5 μL of a 1.5 mol L^–1^ 25H-NBOH solution, 3 μL of solution A and 3 μL of solution B, applied in this order. We performed the PS-MS analysis after 5 min of reaction time.

### 4.5 Electrochemical Measurements

Five successive scans, by cyclic voltammetry (CV), in the potential window from −1.0 V to +1.0 V (vs. Ag) and scan rate of 100 mV s^–1^, were made in supporting electrolyte (BR 0.1 mol L^–1^) for electrochemical conditioning of the SPE-Gr before each measurement. Electrochemical studies were performed by CV at SPE-Gr, using different scan rates and different pH values. The voltammetric detection of NBOHs was optimized using the AdSDPV technique, and the optimal parameters of this technique were obtained by varying the preaccumulation time in the range of 0 to 3 min, the pulse amplitude between 10 and 100 mV, the step potential from 1–10 mV, the pulse width of 10–50 ms and the time interval of 0.1–0.5 s. All electrochemical measurements were performed in triplicate. The optimal parameters were obtained with 1.0 min preaccumulation time, 90 mV pulse amplitude, 7.5 mV step potential, 50 ms pulse width, and 0.5 s time interval. The obtained voltammograms by AdSDPV were subjected to background subtraction using a polynomial fit in the NOVA 2.1 software.

## Supporting Information Available

Additional experiment details, materials, and methods, including PS(+)-MS/MS data of the ion of *m*/*z* 487, a simplified scheme for the hyphenated method, and an interpretation of its results; ^1^H and ^13^C NMR spectra for all compounds synthesized are also included (PDF)
