# Supplementary material for: Colorimetric-Electrochemical Combined Method for the Identification of Drugs of Abuse in Blotter Papers: A Powerful Screening Technique Using Three Analytical Responses
Source: ACS Omega. 2025 Apr 18;10(16):16648–57. doi: 10.1021/acsomega.5c00368 (PMC12044553; doi:10.1021/acsomega.5c00368)
Supplement: Supplementary file 1 — ao5c00368_si_001.pdf [file ao5c00368_si_001.pdf]

Colorimetric-Electrochemical Combined Method for the Identification of Drugs of Abuse in Blotter Papers: A Powerful Screening Using Three Analytical Responses

Cláudia Mancilha Rocha[+,a], Larissa Magalhães de Almeida Melo[+,b], Augusto César Carvalho Santos[a], João Victor Coelho Pimenta[a], Glayton Andrade Souza[b], Luciano Chaves Arantes[c], Wellington Alves de Barros[a], Rodinei Augusti[a], Clésia Cristina Nascentes[a], Wallans Torres Pio dos Santos[*,d], Ângelo de Fátima[*,a]

[a] Departamento de Química, Instituto de Ciências Exatas, Universidade Federal de Minas Gerais, 37270-690, Belo Horizonte, Brazil.
[b] Departamento de Química, Universidade Federal dos Vales do Jequitinhonha e Mucuri, 39100-000, Diamantina, Brazil.
[c] Laboratório de Química e Física Forense, Instituto de Criminalística, Polícia Civil do Distrito Federal, 70610-907, Brasília, Brazil.
[d] Departamento de Farmácia, Universidade Federal dos Vales do Jequitinhonha e Mucuri, 39100-000, Diamantina, Brazil.

+ These authors contributed equally to the work.

*Corresponding Authors:
Email: adefatima.geqob@gmail.com (AF) and wallanst@ufvjm.edu.br (WTPS)

EXPERIMENTAL SECTION

Synthesis and Characterization

The molecules were synthesized following a general procedure and characterized using the methodology outlined in Barros *et al.*, 2021[1].

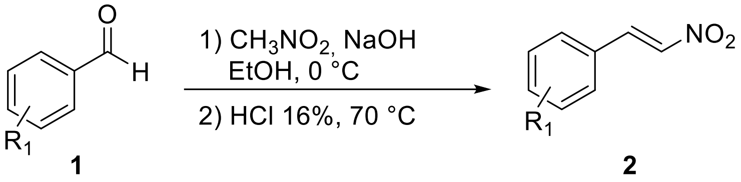

Nitromethane (10 mmol) was slowly added to a mixture of absolute ethanol (100 mL) and the corresponding aldehyde in a 125 mL round-bottom flask. The mixture was kept under stirring at 0 °C. Next, 2.0 mL of a NaOH solution (10.5% w/v) was added dropwise to the mixture under magnetic stirring over 1 hour (this mixture is referred to as Solution 1). In a separate round-bottom flask, 150 mL of 16% (v/v) HCl was added and heated to 70°C while maintaining magnetic stirring. Solution 1 was then slowly added to the heated HCl solution. The reaction was allowed to proceed for 2 hours, after which the solid product was filtered under reduced pressure.

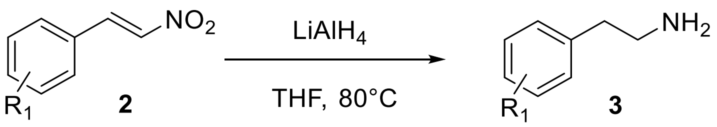

A suspension of $LiAlH_4$ (4 mmol) in dry THF (30 mL) was heated to 75–80 °C under an argon atmosphere. A solution of the compound **2** (1 mmol) and dry THF (10 mL) was then slowly added to the suspension. The reaction was allowed to proceed for 2 hours under continuous magnetic stirring and heating. After completion, the mixture was cooled to room temperature, exposed to air, and sequentially treated with the following additions: cold ether (20 mL), distilled water (152 µL), 15% NaOH (w/v) (152 µL), and finally additional distilled water (455 µL). The mixture was stirred for 5 minutes after each addition. The resulting mixture was dried over MgSO4, filtered, and the solvent was evaporated under reduced pressure. The residue was purified by column chromatography on silica gel using a $CHCl_3$:MeOH (6:1, v/v) solvent system.

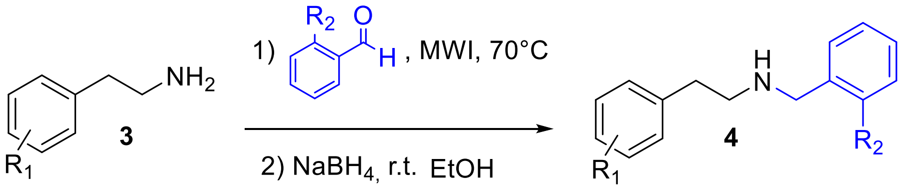

The corresponding aldehyde (1.3 mmol) was added to a mixture of amine **3** (1 mmol) and absolute ethanol (10 mL). The reaction was carried out under microwave irradiation under reflux conditions (25 minutes at 250 W, 70°C). Afterward, crushed $NaBH_4$ (4 mmol) was added to the mixture, and the reaction was allowed to proceed for 2 hours under magnetic stirring at room temperature. The solvent was then evaporated under reduced pressure, and the resulting product was dissolved in 10 mL of CH_2Cl_2. The solution was extracted with water (2 × 10 mL), and the organic phase was collected. The aqueous phase was further extracted with CH_2Cl_2 (3 × 10 mL). The combined organic phases were dried over Na_2SO_4, filtered, and the solvent was evaporated under reduced pressure. The residue was purified by column chromatography on silica gel using a CH_3Cl:MeOH (95:5, v/v) solvent system.

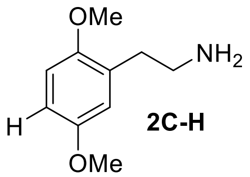 2C-H	**2C-H** ^{1}H NMR ($CDCl_3$, 400 MHz) δ = 6.77 (d, 1H, *J* = 8.6 Hz), 6.73 (dd, 1H, *J* = 6.30 Hz, *J* = 2.8 Hz), 6.70 (d, 1H, *J* = 3.0), 3.76 (s, 3H), 3.75 (s, 3H), 2.92 (t, 2H, *J* = 6.9 Hz), 2.74 (t, 2H, *J* = 6.9 Hz), 2.04 (br, 2H). ^{13}C NMR ($CDCl_3$, 100 MHz) δ = 153.5, 152.1, 129.3, 117.0, 111.5, 111.4, 56.0, 55.8, 42.2, 34.7. The NMR data are consistent with those previously reported in the literature [1,2].
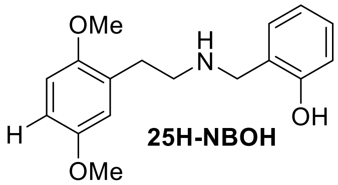 25H-NBOH	**25H-NBOH** 1H NMR ($CDCl_3$, 400 MHz) δ = 7.14 (td, 1H, *J* = 7.8 and 1.6 Hz), 6.96 (d, 1H, *J* = 7.5 Hz), 6.81 (dd, 1H, *J* = 8.0 and 0.9 Hz), 6.79-6,71 (m, 4H), 3.98 (s, 2H), 3.76 (s, 3H), 3.75 (s, 3H), 2.93-2.90 (m, 2H), 2.85-2.82 (m, 2H). ^{13}C NMR ($CDCl_3$, 100 MHz) δ = 158.6, 153.6, 152.0, 128.8, 128.7, 128.4, 122.7, 119.0, 116.9, 116.5, 111.8, 111.5, 56.0, 55.8, 52.6, 48.5, 30.5. The NMR data are consistent with those previously reported in the literature [1].
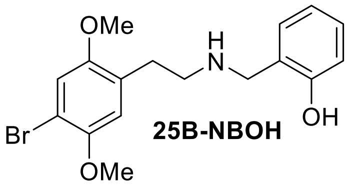 25B-NBOH	**25B-NBOH** ^{1}H NMR ($CDCl_3$, 400 MHz) δ = 7.15 (dt, 1H, *J* = 7.8 and 1.7 Hz), 7.03 (*s*, 1H), 6.96 (dd, 1H, *J* = 7.4 and 1.6 Hz), 6.81 (dd, 1H, *J* = 8.0 and 1.2 Hz), 6.76 (dt, 1H, *J* = 7.6 and 1.2 Hz), 6.72 (s, 1H), 3.98 (s, 2H), 3.84 (s, 3H), 3.76 (s, 3H), 2.92-2.88 (m, 2H), 2.83-2.79 (m, 2H). ^{13}C NMR ($CDCl_3$, 100 MHz) δ = 158.4, 152.1, 150.1, 128.8, 128.4, 127.9, 122.6, 119.1, 116.5, 116.1, 115.0, 109.5, 57.1, 56.2, 52.7, 48.3, 30.7. The NMR data are consistent with those previously reported in the literature [1].
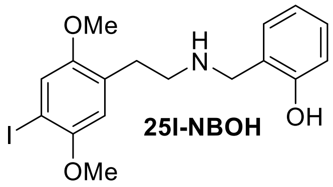 25I-NBOH	**25I-NBOH** ^{1}H NMR ($CDCl_3$, 400 MHz) δ = 7.22 (s, 1H), 7.15 (dt, 1H, *J* = 7.6 and 1.6 Hz), 6.96 (d, 1H, *J* = 7.4 Hz), 6.82 (dd, 1H, *J* = 8.2 and 0.8 Hz), 6.76 (dt, 1H, *J* = 7.4 and 0.8 Hz), 6.64 (s, 1H), 3.98 (s, 2H), 3.82 (s, 3H), 3.76 (s, 3H), 2.92-2.88 (m, 2H), 2.83-2.80 (m, 2H). ^{13}C NMR ($CDCl_3$, 100 MHz) δ = 158.5, 152.7, 152.5, 129.0, 128.8, 128.4, 122.6, 121.8, 119.1, 116.5, 113.8, 83.1, 57.3, 56.2, 52.7, 48.3, 30.9. The NMR data are consistent with those previously reported in the literature [1,3].
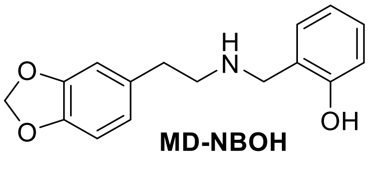 MD-NBOH	**MD-NBOH** ^{1}H NMR ($CDCl_3$, 400 MHz) δ = 7.16 (t, 1H, *J* = 8 Hz), 6.96 (d, 1H, *J* = 4 Hz), 6.83 (d, 1H, *J* = 8 Hz), 6.80-6.60 (m, 4H), 5.92 (s, 2H), 4.58 (s, NH), 3.97 (s, 2H), 2.95-2.85 (m, 2H), 2.81-2.72 (m, 2H). ^{13}C NMR ($CDCl_3$, 100 MHz) δ = 158.2, 147.9, 146.7, 132.6, 128.8, 122.3, 122.3, 121.6, 119.4, 116.6, 108.9, 108.4, 100.9, 52.4, 49.6, 35.3. The NMR data are consistent with those previously reported in the literature [4].
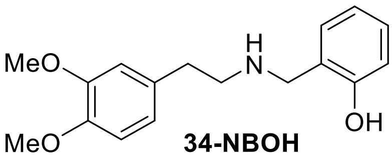 34-NBOH	**34-NBOH** ^{1}H NMR ($CDCl_3$, 400 MHz) δ = 7.16 (t, 1H, *J* = 8 Hz, *J* = 1,4 Hz), 6.98 (s, 1H), 6.86-6.68 (m, 5H), 3.97 (s, 2H), 3.87 (s, 3H), 3.85 (s, 3H), 2.96-2.89 (m, 2H), 2.83-2.75 (m, 2H). ^{13}C NMR ($CDCl_3$, 100 MHz) δ = 158.3, 149.1, 147.7, 131.5, 128.7, 128.3, 122.4, 120.6, 119.0, 116.4, 111.9, 111.4, 56.0, 55.9, 52.6, 49.7, 35.4. The NMR data are consistent with those previously reported in the literature [4].
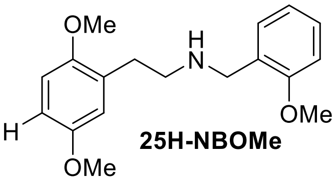 25H-NBOMe	**25H-NBOMe** ^{1}H NMR ($CDCl_3$, 400 MHz) δ = 7.24-7.20 (m, 2H), 6.89 (t, 1H, *J* = 7.4 Hz), 6.82 (d, 1H, *J* = 8.2 Hz), 6.75 (d, 1H, *J* = 8.8 Hz), 6.74 (d, 1H, *J* = 2.8 Hz), 6.70 (dd, 1H, *J* = 8.8 and 2.9 Hz), 3.84 (s, 2H), 3.75 (s, 3H), 3.74 (s, 3H), 3.72 (s, 3H), 2.96 (br), 2.89-2.83 (m, 4H). ^{13}C NMR ($CDCl_3$, 100 MHz) δ = 157.6, 153.4, 151.9, 130.0, 129.4, 128.4, 127.4, 120.4, 116.6, 111.4, 111.3, 110.1, 55.9, 55.6, 55.1, 48.9, 48.5, 30.6. The NMR data are consistent with those previously reported in the literature [1,2].

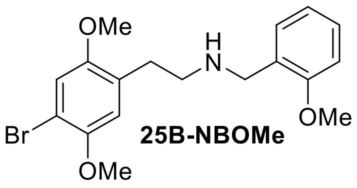 25B-NBOMe	**25B-NBOMe** ^{1}H NMR ($CDCl_3$, 400 MHz) δ = 7.25-7.19 (m, 2H), 7.00 (s, 1H), 6.90 (t, 1H, J = 7.3 Hz), 6.82 (d, 1H, J = 8.0 Hz), 6.76 (s, 1H), 3.83 (s, 2H), 3.81 (s, 3H), 3.74 (s, 3H), 3.71 (s, 3H), 2.88 – 2.80 (m, 4H), 2.76 (br). ^{13}C NMR ($CDCl_3$, 100 MHz) δ = 157.7, 151.2, 150.0, 130.2, 128.6, 128.5, 127.2, 120.5, 116.0, 115.1, 110.3, 109.1, 57.1, 56.2, 55.3, 49.1, 48.3, 30.7. The NMR data are consistent with those previously reported in the literature [1,5].
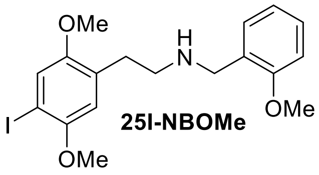 25I-NBOMe	**25I-NBOMe** ^{1}H NMR ($CDCl_3$, 400 MHz) δ = 7.25-7.19 (m, 2H), 7.18 (s, 1H), 6.89 (t, 1H, J = 7.5 Hz), 6.82 (d, 1H, J = 8.1 Hz), 6.68 (s, 1H), 3.83 (s, 2H), 3.80 (s, 3H), 3.73 (s, 3H), 3.70 (s, 3H), 2.91 – 2.77 (m, 4H), 2.47 (br). ^{13}C NMR ($CDCl_3$, 100 MHz) δ = 157.6, 152.5, 152.4, 130.1, 129.5, 128.5, 127.0, 121.6, 120.4, 113.8, 110.2, 82.5, 57.1 56.1, 55.2, 49.0, 48.1, 30.7. The NMR data are consistent with those previously reported in the literature [1].

Colorimetric Solutions Preparation

- **Solution 1 –** Aqueous solution of potassium ferricyanide ($K_3[Fe(CN)_6]$) 8.0% (w/v): To a 100 mL volumetric flask, was added 8 g of $K_3[Fe(CN)_6]$, then deionized water (resistivity 18.2 MΩ cm) was added to the marked line.
- **Solution 2 –** NH_4OH/NH_4Cl buffer solution (pH = 10): To a 100 mL volumetric flask, was added 6.76 g of NH_4Cl salt and 57.2 mL of NH_4OH concentrate solution (about 30%), then deionized water (resistivity 18.2 MΩ cm) was added to the marked line.

The solutions **1** and **2** were stored separately for up to one year and mixed in a proportion of 2:13 (v/v) – the use of the resulting solution (**A**) is recommended up to a month after the preparation.

- **Solution B –** Aqueous solution of 4-aminoantipyrine (4-AAP) 0.67% (w/m): To a 100 mL volumetric flask, was added 0.67 g of 4-aminoantipyrine, then deionized water (resistivity 18.2 MΩ cm) was added to the marked line – the use of the resulting solution (**B**) is recommended up to a month after the preparation.

FIGURES AND TABLES

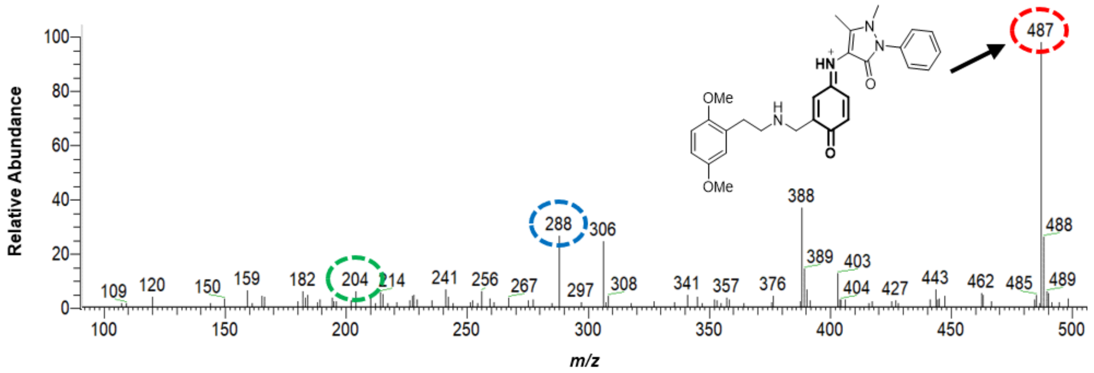

Figure S1. PS(+)-MS of the on-surface reaction between 25H-NBOH and 4-AAP catalyzed by $K_3[Fe(CN)_6]$ after 5 minutes.

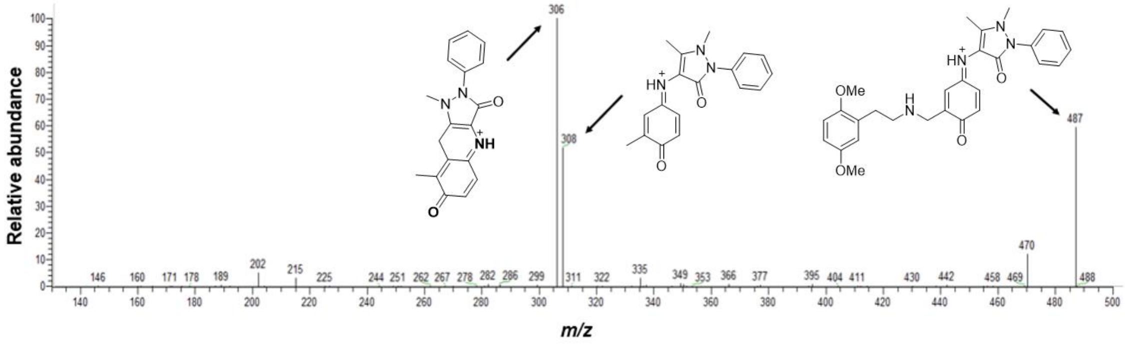

Figure S2. PS(+)-MS/MS of the ion of *m/z* 487.

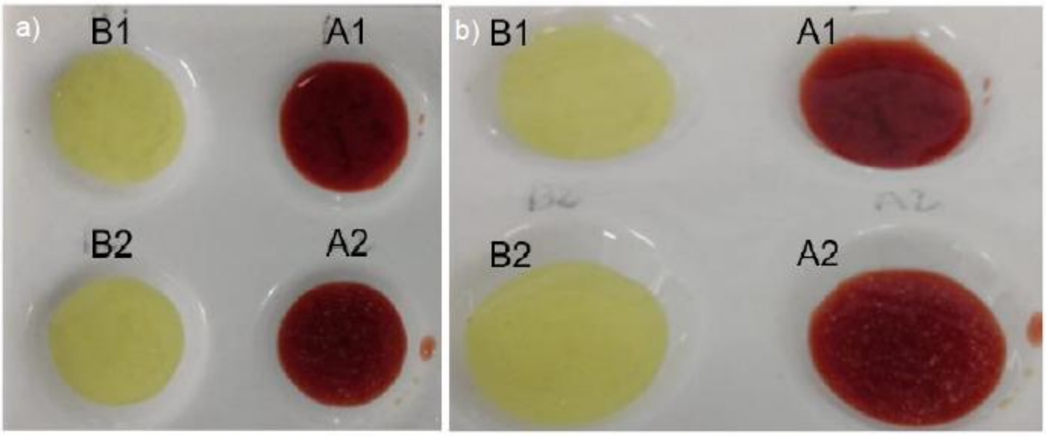

Figure S3. The blank solution (B) and 25H-NBOH (A). B1 and A1 refer to the addition of 4-AAP followed by $K_3[Fe(CN)_6]$, and B2 and A2 refer to the addition of $K_3[Fe(CN)_6]$ followed by 4-AAP. The record b) focuses on the insoluble crystals formed.

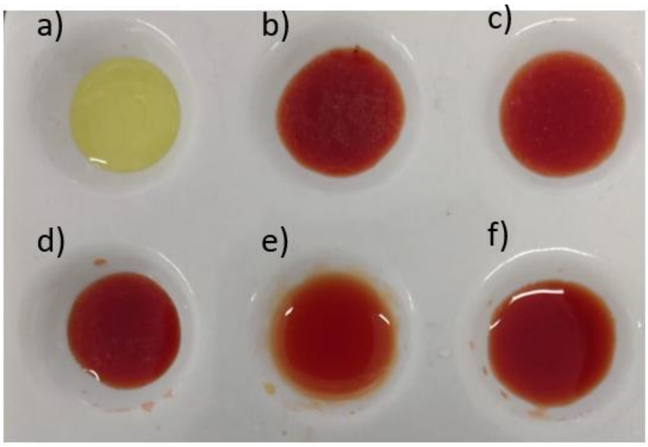

Figure S4. Colors observed for the a) blank and metanolic solutions (1 mg mL^{-1}) for b) 25H-NBOH, c) 25B-NBOH, d) 25I-NBOH, e) MD-NBOH, and f) 34-NBOH.

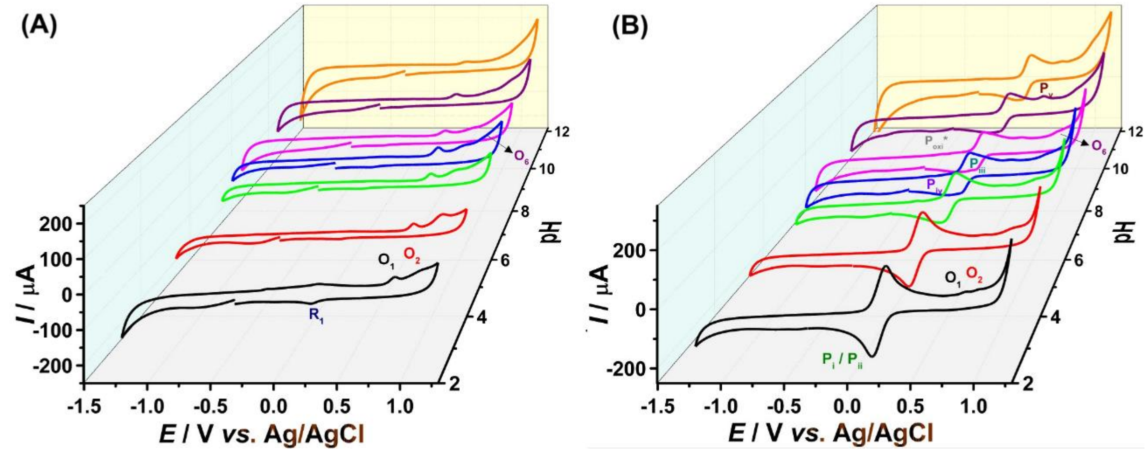

Figure S5. 3D plots of recorded cyclic voltammograms (first scan) of 0.4 × 10^{-3} mol L^{-1} 25H-NBOH in 0.1 mol L^{-1} BR buffer solution with different pH values (from 2.0 to 12.0) in absence (A) and presence of the CRs (B). All potential sweeps started at –0.3 V (*vs*. Ag/AgCl), with a scan rate of 50 mV s^{-1}.

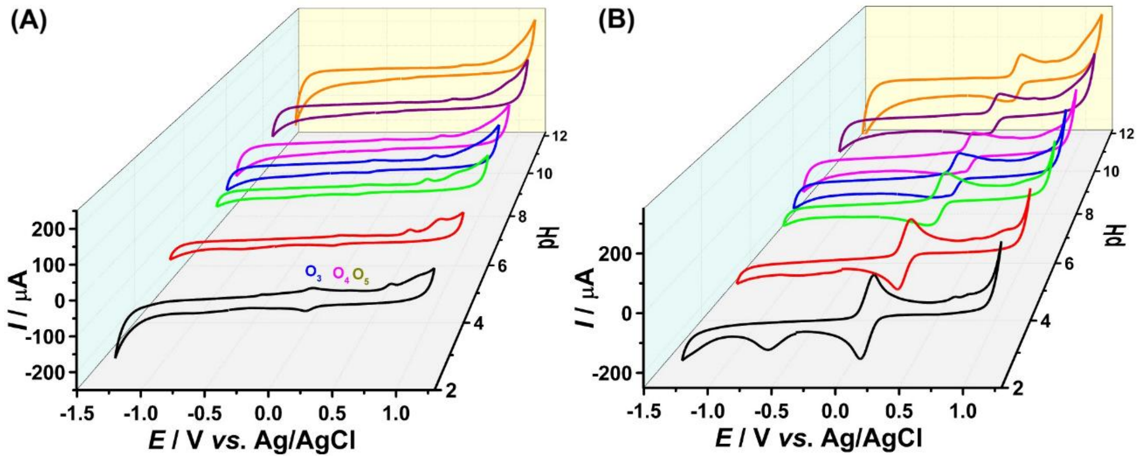

Figure S6. 3D plots of recorded cyclic voltammograms (second scan) of 0.4 × 10^{-3} mol L^{-1} 25H-NBOH in 0.1 mol L^{-1} BR buffer solution with different pH values (from 2.0 to 12.0) in absence (**A**) and presence of the CRs (**B**). All potential sweeps started at –0.3 V (*vs*. Ag/AgCl), with a scan rate of 50 mV s^{-1}.

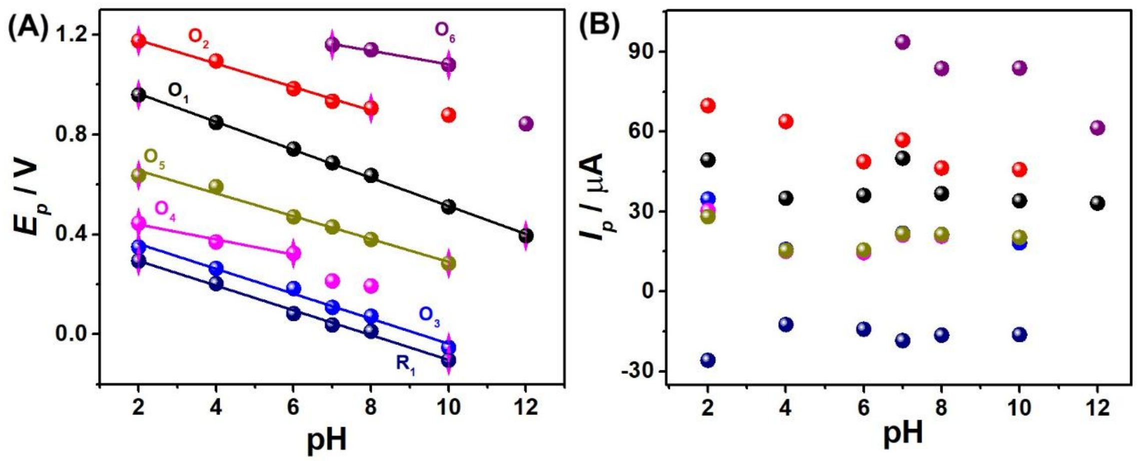

Figure S7. (**A**) Relationship between E_p and pH, and (**B**) relationship between i_p and pH for the redox processes of 25H-NBOH, data obtained from Figure 2A and Figure S3 A. Black dots represent O_1, red dots represent O_2, blue dots represent O_3, pink dots represent O_4, yellow dots represent O_5, purple dots represent O_6, and dark blue dots represent R_1.

Table S1. Linear relationship between E_p and pH for 25H-NBOH.

Redox Process	pH range	Linear regression equation	R^2
O_1	2.0 – 12.0	**(1S)** E_p (V) = 1.08 (±0.01) – 0.056 (±0.001) × *pH*	0.999
O_2	2.0 – 8.0	**(2S)** E_p (V) = 1.27 (±0.01) – 0.047 (±0.002) × *pH*	0.993
O_3	2.0 – 10.0	**(3S)** E_p (V) = 0.46 (±0.02) – 0.050 (±0.002) × *pH*	0.991
O_4	2.0 – 6.0	**(4S)** E_p (V) = 0.50 (±0.02) – 0.030 (±0.004) × *pH*	0.980
O_5	2.0 – 10.0	**(5S)** E_p (V) = 0.75 (±0.02) – 0.046 (±0.002) × *pH*	0.987
O_6	7.0 – 10.0	**(6S)** E_p (V) = 1.36 (±0.02) – 0.027 (±0.003) × *pH*	0.992
R_1	2.0 – 10.0	**(7S)** E_p (V) = 0.39 (±0.01) – 0.050 (±0.002) × *pH*	0.994

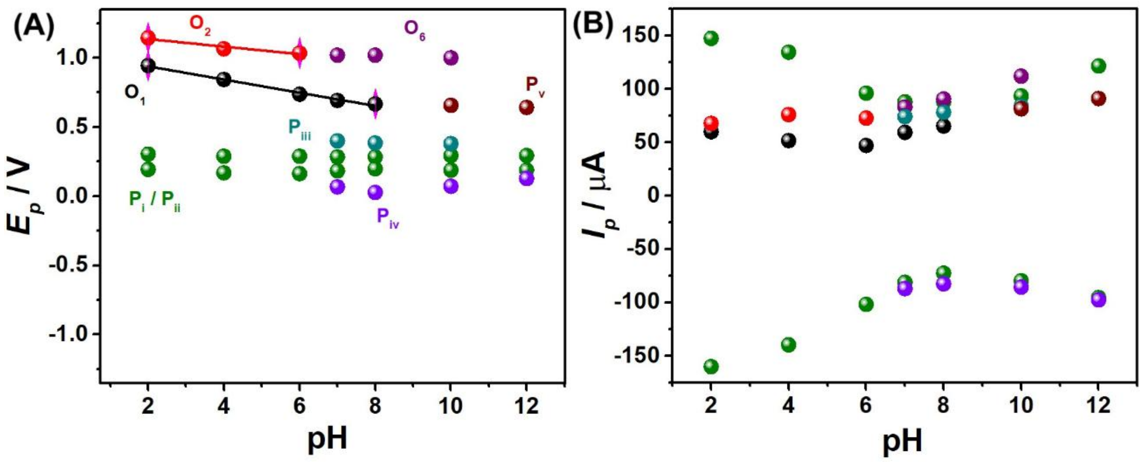

Figure S8. (**A**) Relationship between E_p and pH, and (**B**) relationship between i_p and pH for the redox processes of 25H-NBOH in presence of CR, data obtained from Figure 2B and Figure S3 B. Black dots represent O_1, red dots represent O_2, purple dots represent O_6, green dots represent P_I/P_{II}, aqua dots represent P_{III}, lilac dots represent P_{IV}, and brown dots represent P_V.

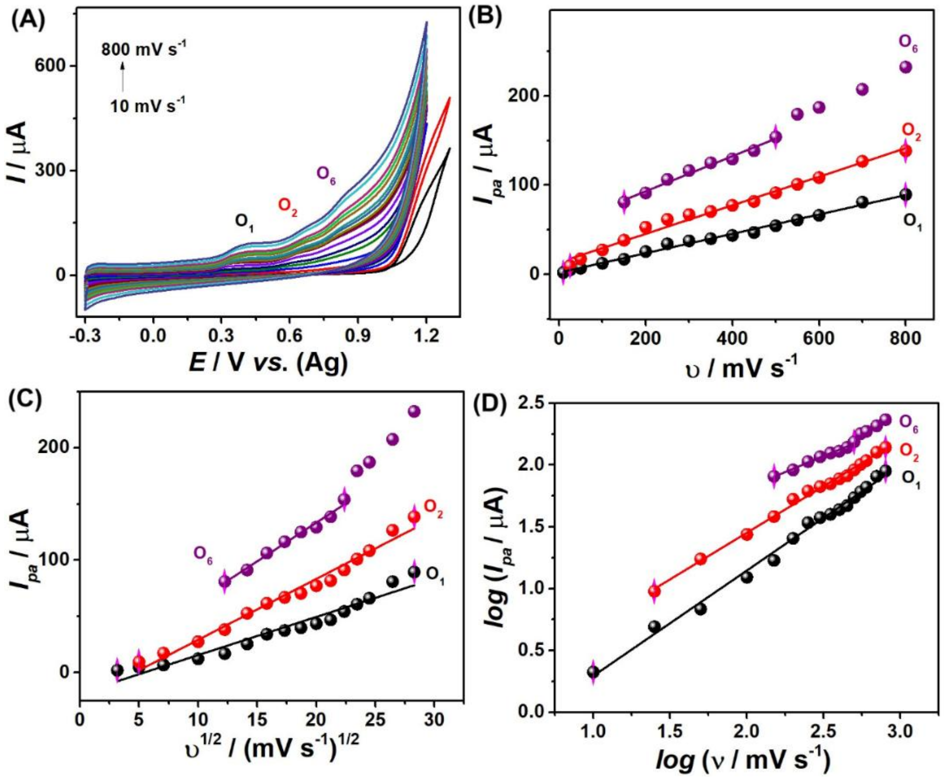

Figure S9. (A) Cyclic voltammograms at scan rates ranging from 10 to 800 mV s^{-1} on SPE-Gr for 0.4 × 10^{-3} mol L^{-1} 25H-NBOH in 0.1 mol L^{-1} BR buffer solution at pH 10.0. Plots of the linear regressions obtained for (**B**) I_{pa} *vs. v*; (**C**) I_{pa} *vs.* v^2, and (**D**) log I_{pa} *vs.* log *v* are also shown.

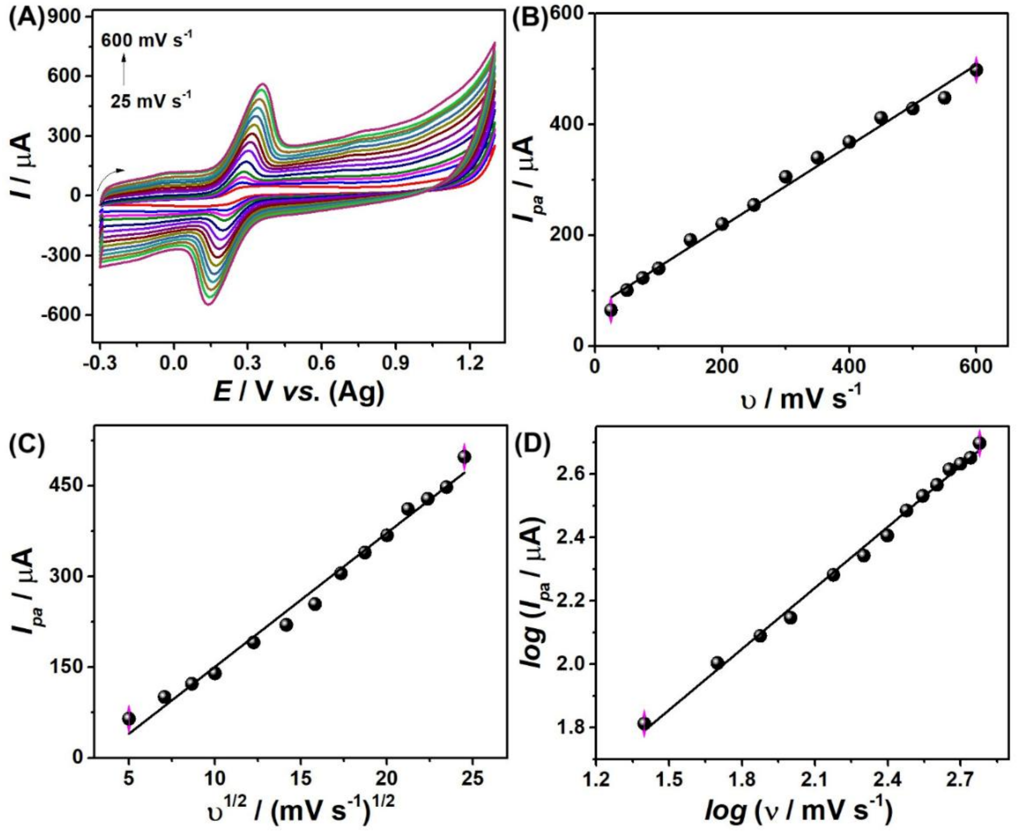

Figure S10. (**A**) Cyclic voltammograms at scan rates ranging from 25 to 600 mV s^{-1} on SPE-Gr for 0.4 × 10^{-3} mol L^{-1} 25H-NBOH with CR in 0.1 mol L^{-1} BR buffer solution at pH 10.0. Plots of the linear regressions obtained for (**B**) I_{pa} *vs.* v; (**C**) I_{pa} *vs.* v^2, and (**D**) log I_{pa} *vs.* log v are also shown.

Table S1. Linear regression equations and R^2 values of the plots of log I_p *vs.* log v.

Electrochemical process	Linear regression equation (*log Ip vs. log v*)	R^2
O_1	**(8S)** *log* I_{pa} (μA) = – 0.56 (± 0.05) + 0.85 (± 0.02) × *log* v (mV s^{-1})	0.99
O_2	**(9S)** *log* I_{pa} (μA) = – 0.06 (± 0.03) + 0.76 (± 0.01) × *log* v (mV s^{-1})	0.99
O_6	**(10S)** *log* I_{pa} (μA) = + 0.78 (± 0.05) + 0.53 (± 0.02) × *log* v (mV s^{-1})	0.99
P_{oxi}*	**(11S)** *log* I_{pa} (μA) = + 0.89 (± 0.02) + 0.64 (± 0.01) × *log* v (mV s^{-1})	0.99

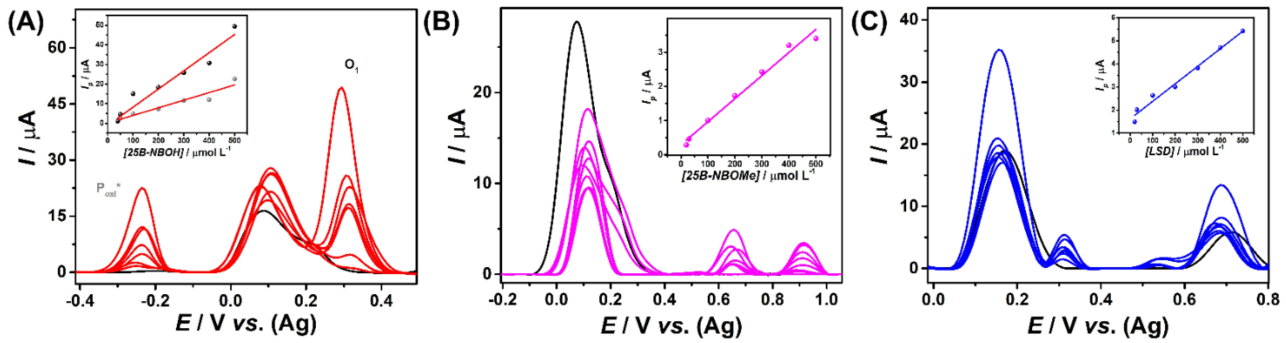

Figure S11. (**A**) AdSDPVs voltammograms of CR before and after addition of [25B-NBOH] in 0.1 mol L^{-1} BR pH 10. [25B-NBOH]: 50, 100; 200; 300; 400 and 500 µmol L^{-1}; Inserted linear regressions I_p *vs*. [*25B-NBOH*]. (**B**) AdSDPVs voltammograms of CR before and after addition of [25I-NBOMe] in 0.1 mol L^{-1} BR pH 10. [25I-NBOMe]: 10, 20; 30; 100; 200; 300; 400 and 500 µmol L^{-1}; Inserted linear regressions I_p *vs*. [*25I-NBOMe*]. (**C**) AdSDPVs voltammograms of CR before and after addition of [LSD] in 0.1 M BR pH 10. [LSD]: 20; 30; 100; 200; 300; 400 and 500 µmol L^{-1}; Inserted linear regressions I_p *vs*. [*LSD*].

Table S3. Linear regression equations and R^2 values of the plots of I_p *vs*. [*Analyte*] obtained from Figure S11.

Drug	Linear range (µmol L^{-1})	Linear regression	R^2
25B-NBOH	40 – 500	**(12S)** I_p (µA) = -1.1 (±0.2) + 0.093 (±0.007) × [*NBOH*] (µmol L^{-1})	0.970
	40 – 500	**(13S)** I_p (µA) = 0.1 (±0.1) + 0.039 (±0.005) × [*NBOH*] (µmol L^{-1})	0.926
25I-NBOMe	20 – 500	**(14S)** I_p (µA) = 0.29 (±0.1) + 0.0068 (±0.0004) × [*NBOMe*] (µmol L^{-1})	0.984
LSD	20 – 500	**(15S)** I_p (µA) = 1.6 (±0.1) + 0.0076 (±0.0004) × [*LSD*] (µmol L^{-1})	0.984

Table S4. Results of analyzes of some of the real samples by combined method (colorimetric-electrochemical – screening test) and confirmatory method, provided by PCDF.

Seized Samples	Screening Test	Confirmatory Method (GC-MS)
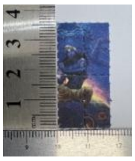	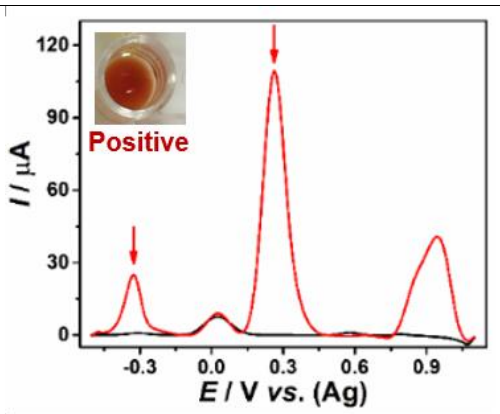	25C-NBOH
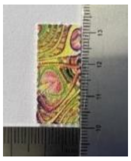	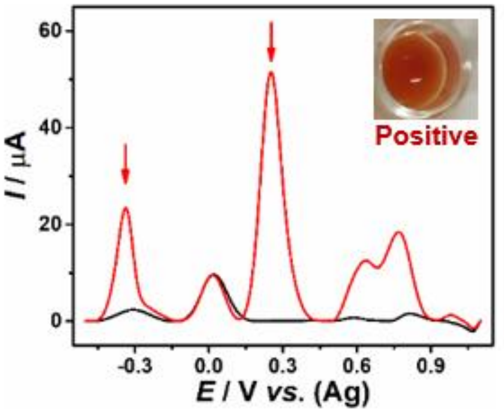	25E-NBOH
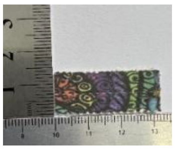	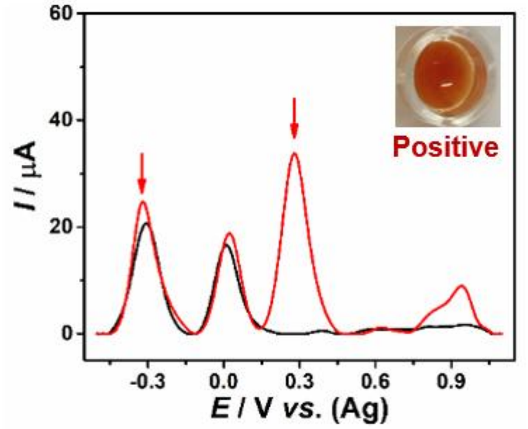	25I-NBOH

<table>
<tr>
<td>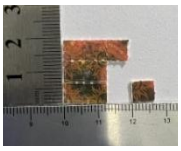</td>
<td>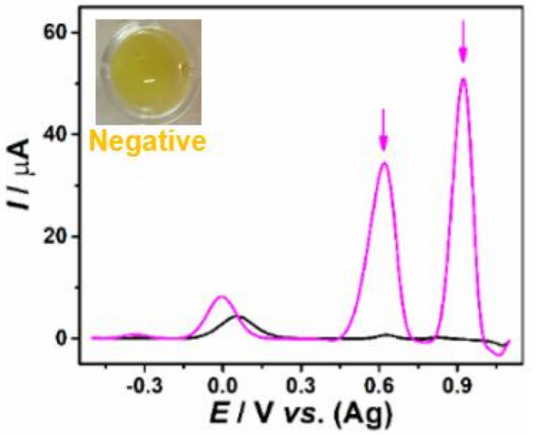
</td>
<td>25B-NBOMe</td>
</tr>
<tr>
<td>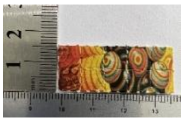</td>
<td>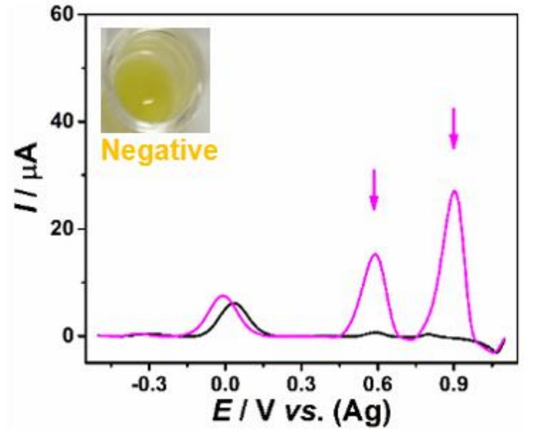
</td>
<td>25C-NBOMe</td>
</tr>
<tr>
<td>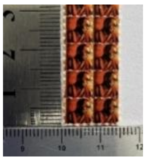</td>
<td>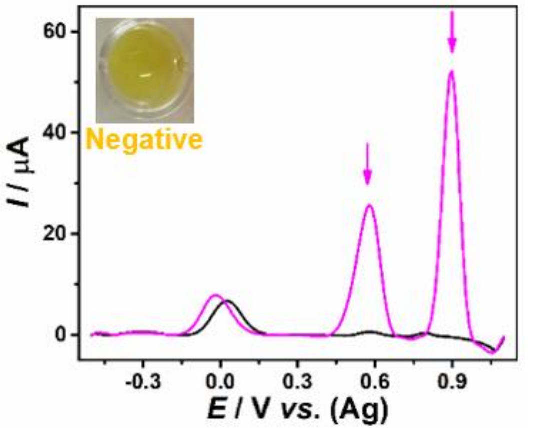
</td>
<td>25I-NBOMe</td>
</tr>
<tr>
<td>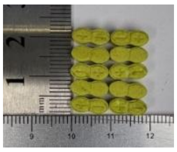</td>
<td>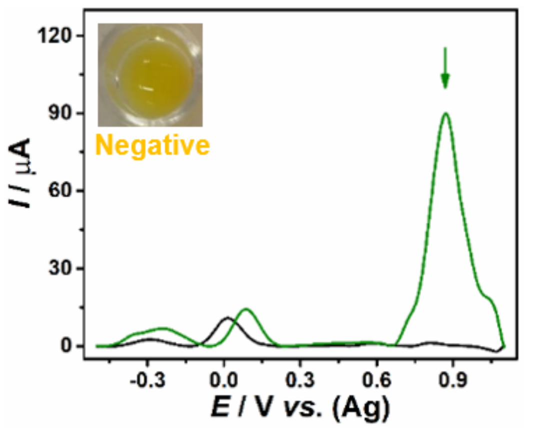
</td>
<td>2C-B</td>
</tr>
</table>

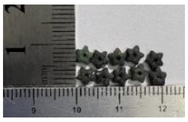	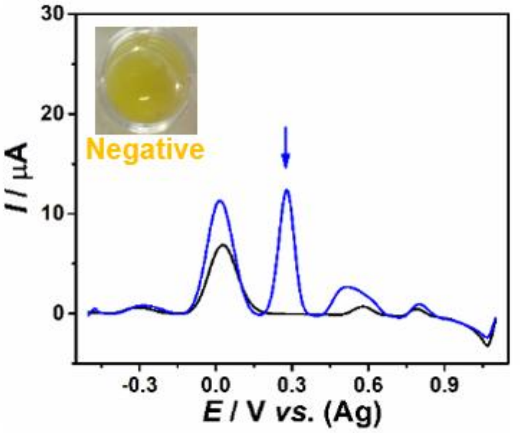Negative<br>I / µA<br>E / V vs. (Ag)<br>30<br>20<br>10<br>0<br>-0.3<br>0.0<br>0.3<br>0.6<br>0.9	LSD
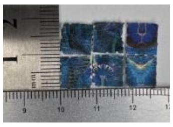	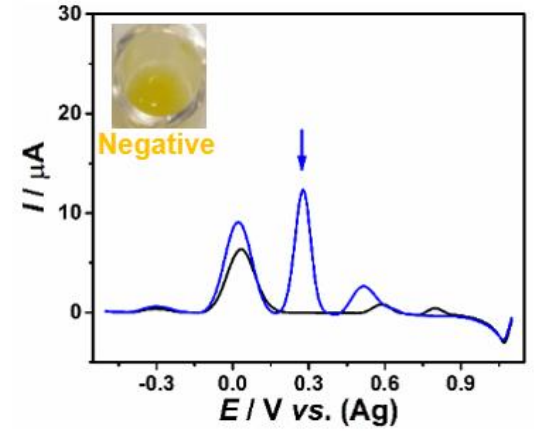Negative<br>I / µA<br>E / V vs. (Ag)<br>30<br>20<br>10<br>0<br>-0.3<br>0.0<br>0.3<br>0.6<br>0.9	LSD
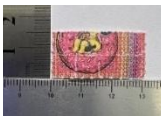	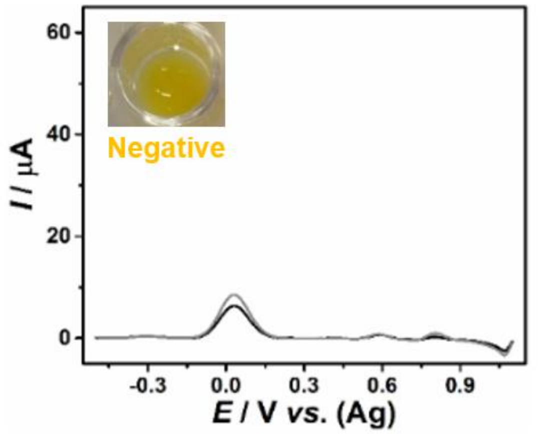Negative<br>I / µA<br>E / V vs. (Ag)<br>60<br>40<br>20<br>0<br>-0.3<br>0.0<br>0.3<br>0.6<br>0.9	ND

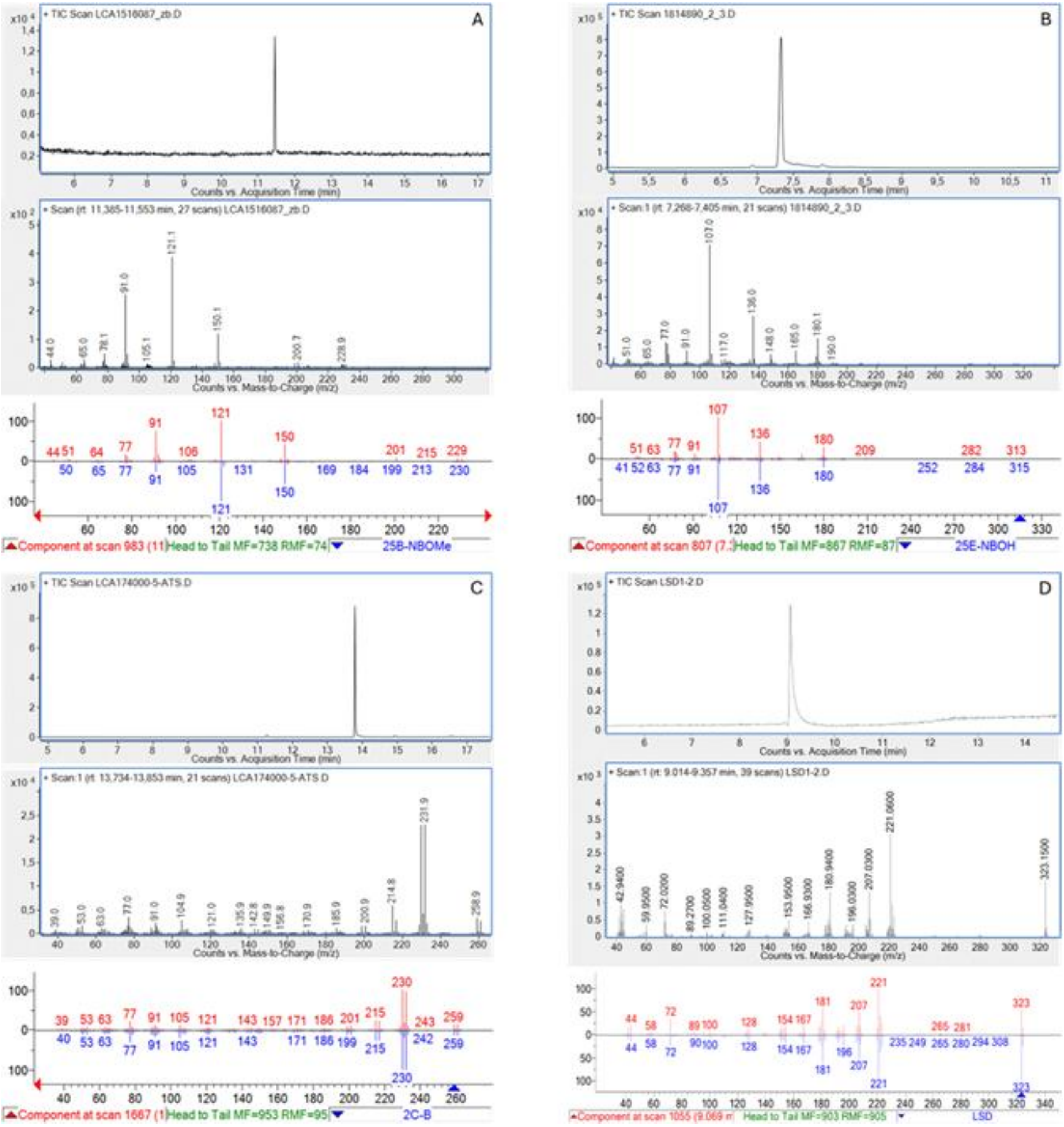

Figure S12. Total ion chromatograms (TIC), mass spectra (MS), and MS library comparisons for seized samples containing 25B-NBOMe (**A**), 25E-NBOH (**B**), 2C-B (**C**), and LSD (**D**).

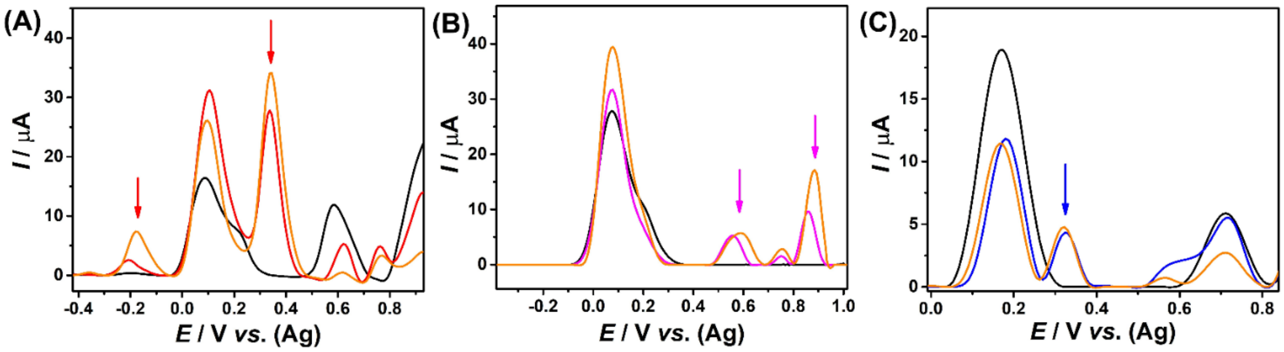

Figure S13. AdSDPVs voltammograms recorded in 0.1 mol L^{-1} BR buffer solution pH 10.0 on SPE-Gr for seized samples containing 25B-NBOH (red) (**A**), 25I-NBOMe (magenta) (**B**), and LSD (blue) (**C**), before and after addition of 85 µmol L^{-1} of each standard, respectively (orange lines). In black, the result of the control negative and the other results after the colorimetric reaction.

NMR SPECTRA

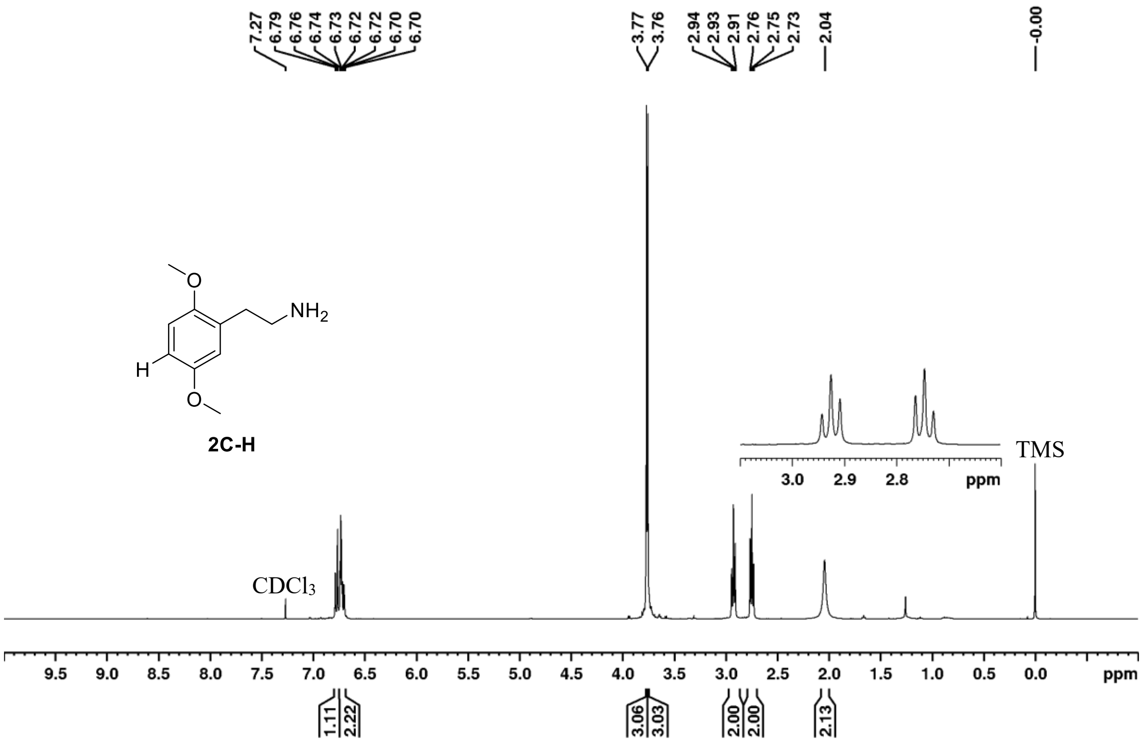

Figure S14. ^{1}H NMR (400 MHz, $CDCl_3$) of 2C-H.

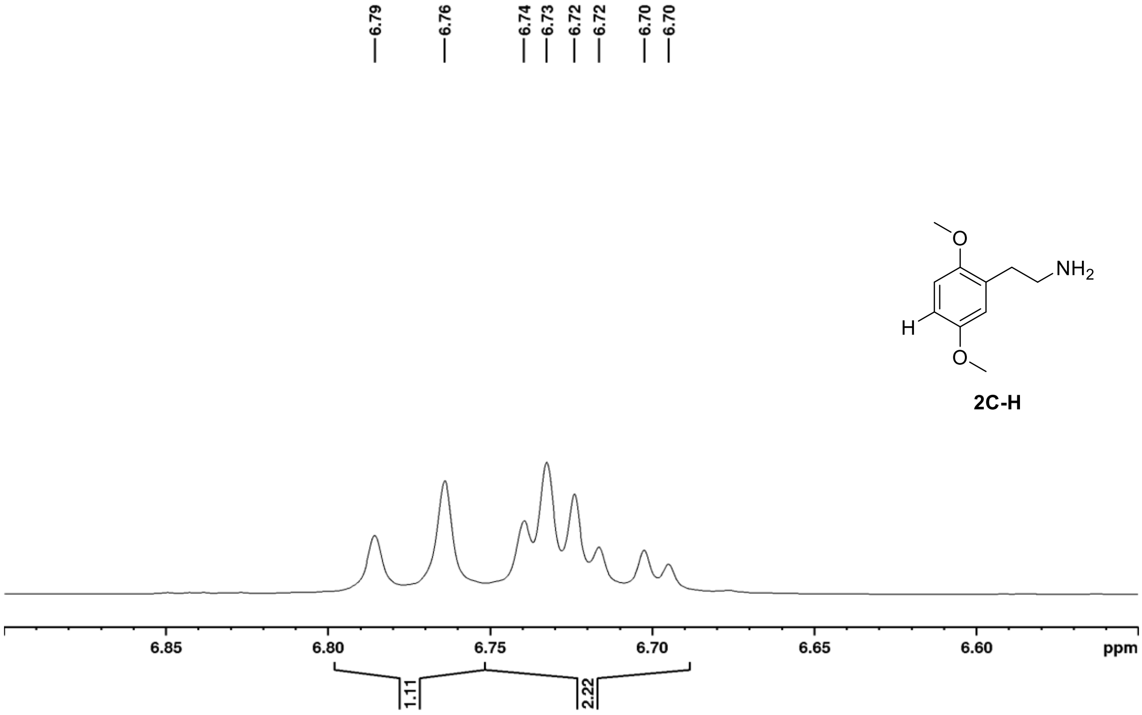

Figure S15. ^{1}H NMR (400 MHz, $CDCl_3$) of 2C-H – Expanded.

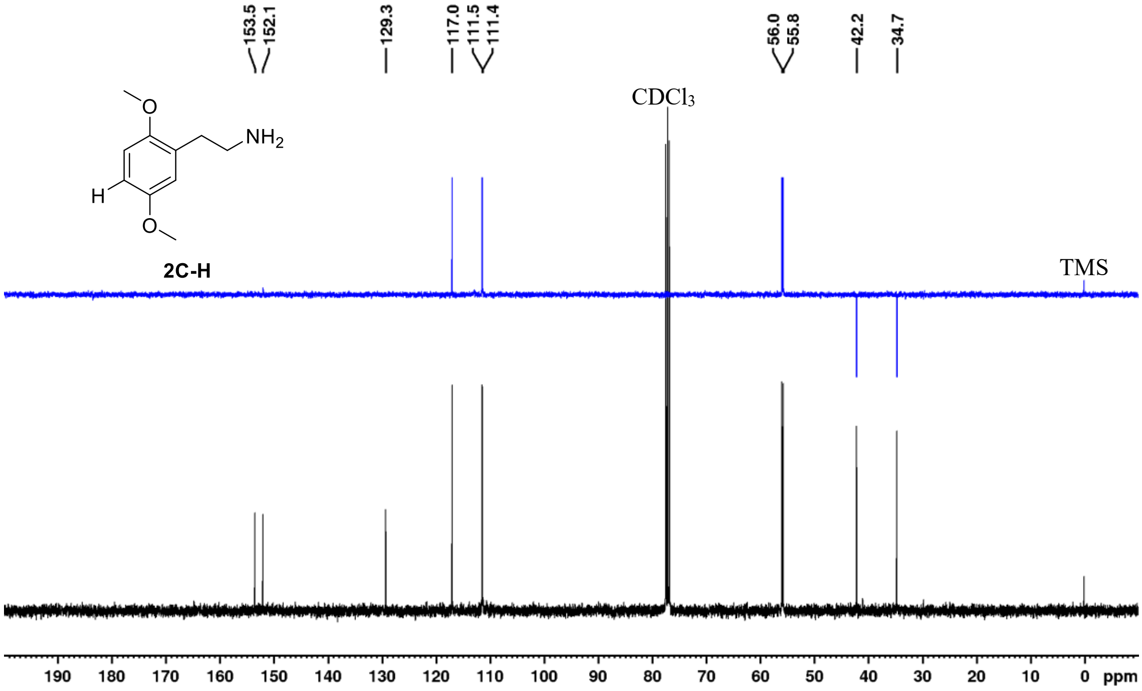

Figure S16. ^{13}C NMR (100 MHz, $CDCl_3$) of 2C-H.

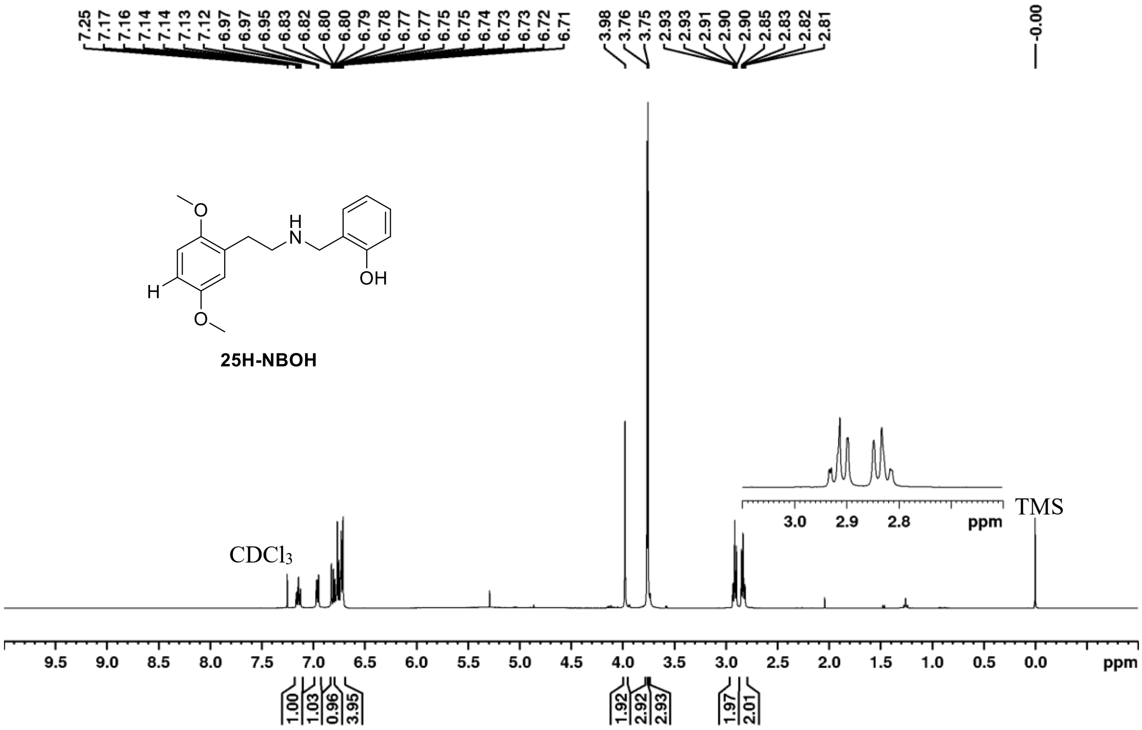

Figure S17. ^{1}H NMR (400 MHz, $CDCl_3$) of 25H-NBOH.

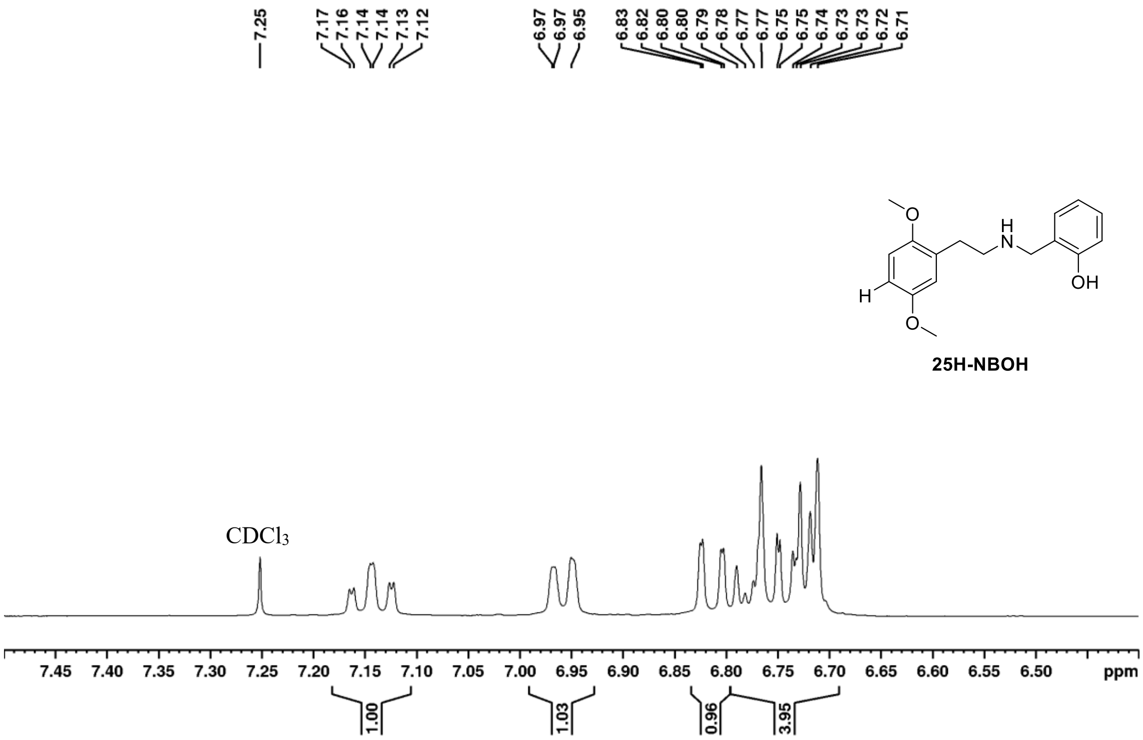

Figure S18. ^{1}H NMR (400 MHz, $CDCl_3$) of 25B-NBOH – Expanded.

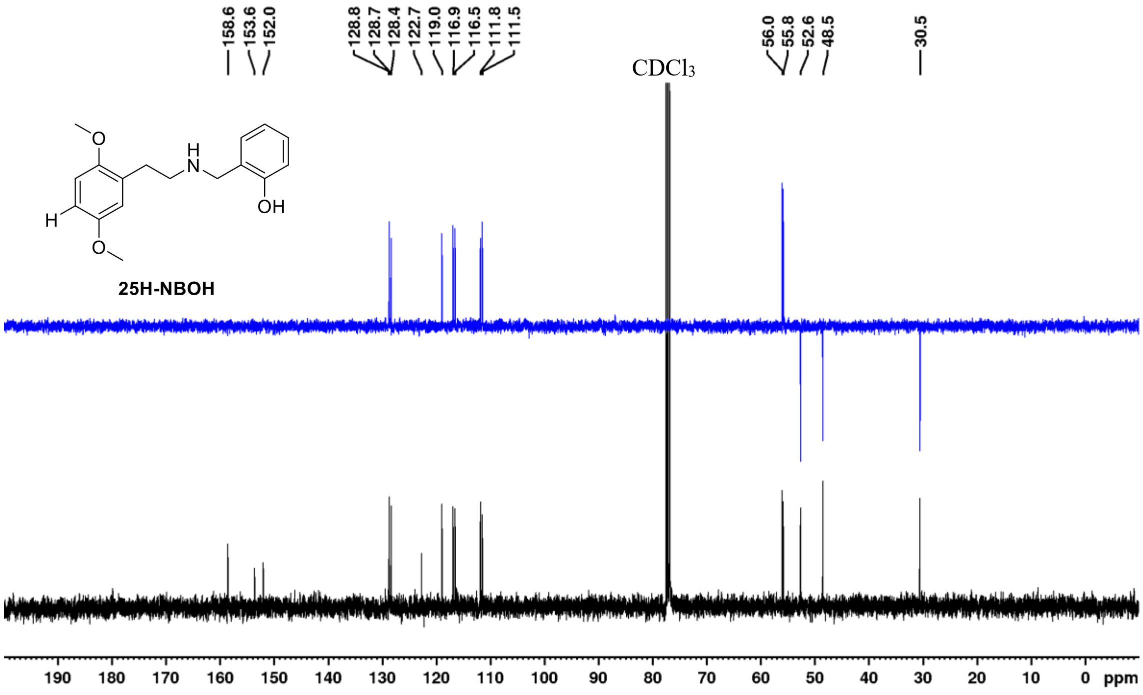

Figure S19. ^{13}C NMR (100 MHz, $CDCl_3$) of 25H-NBOH.

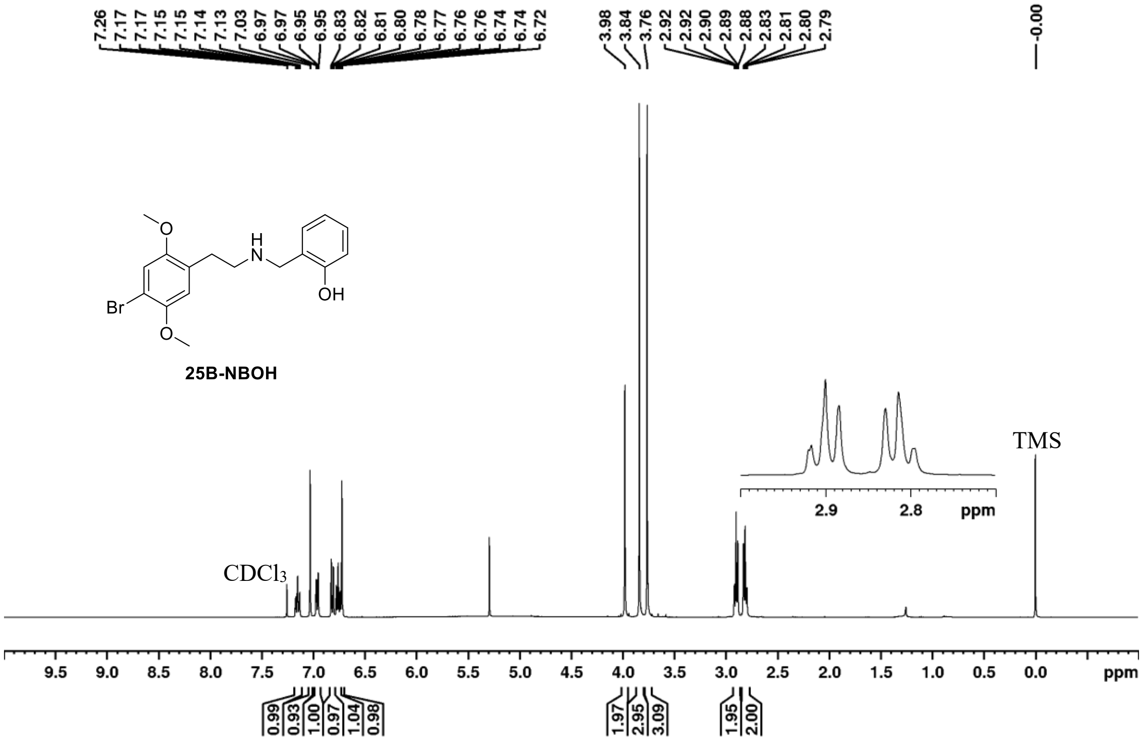

Figure S20. ^{1}H NMR (400 MHz, $CDCl_3$) of 25B-NBOH.

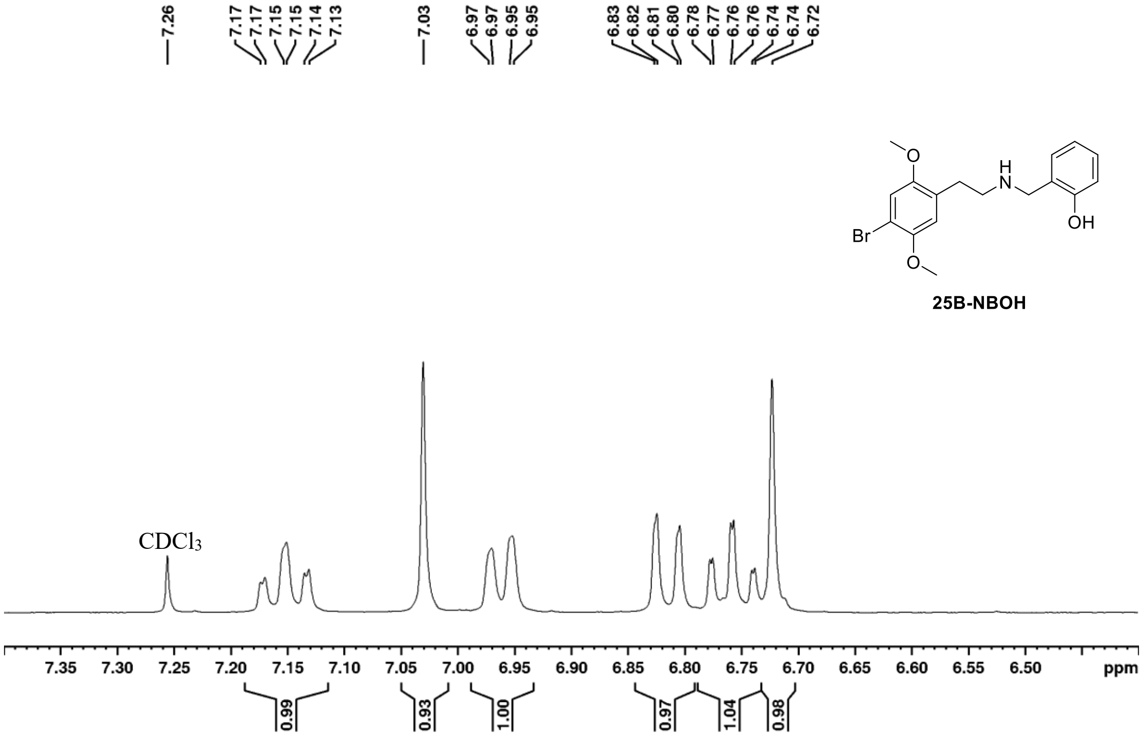

Figure S21. ^{1}H NMR (400 MHz, $CDCl_3$) of 25B-NBOH – Expanded.

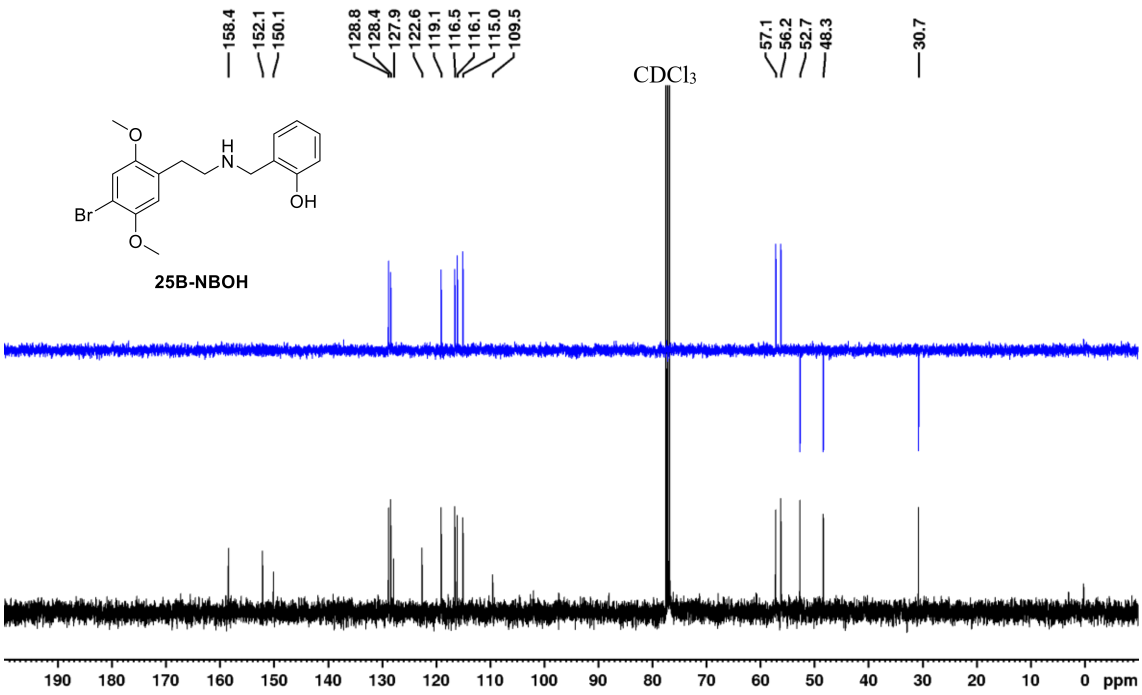

Figure S22. ^{13}C NMR (100 MHz, $CDCl_3$) of 25B-NBOH.

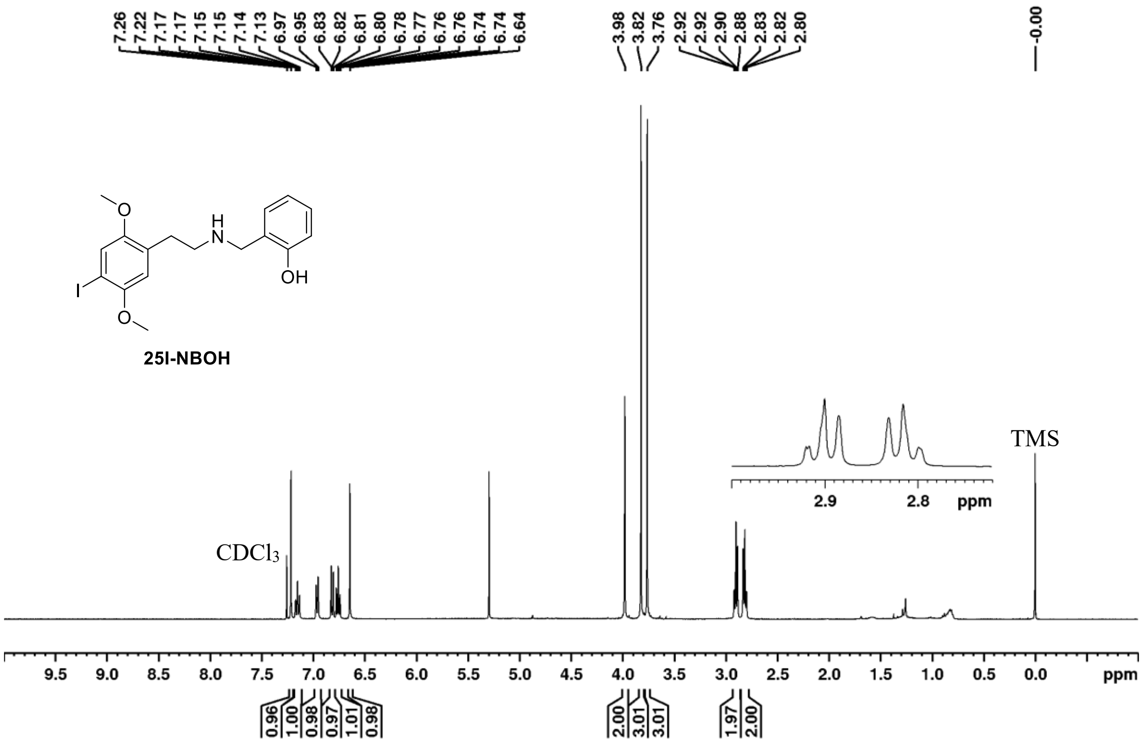

Figure S23. ^{1}H NMR (400 MHz, $CDCl_3$) of 25I-NBOH.

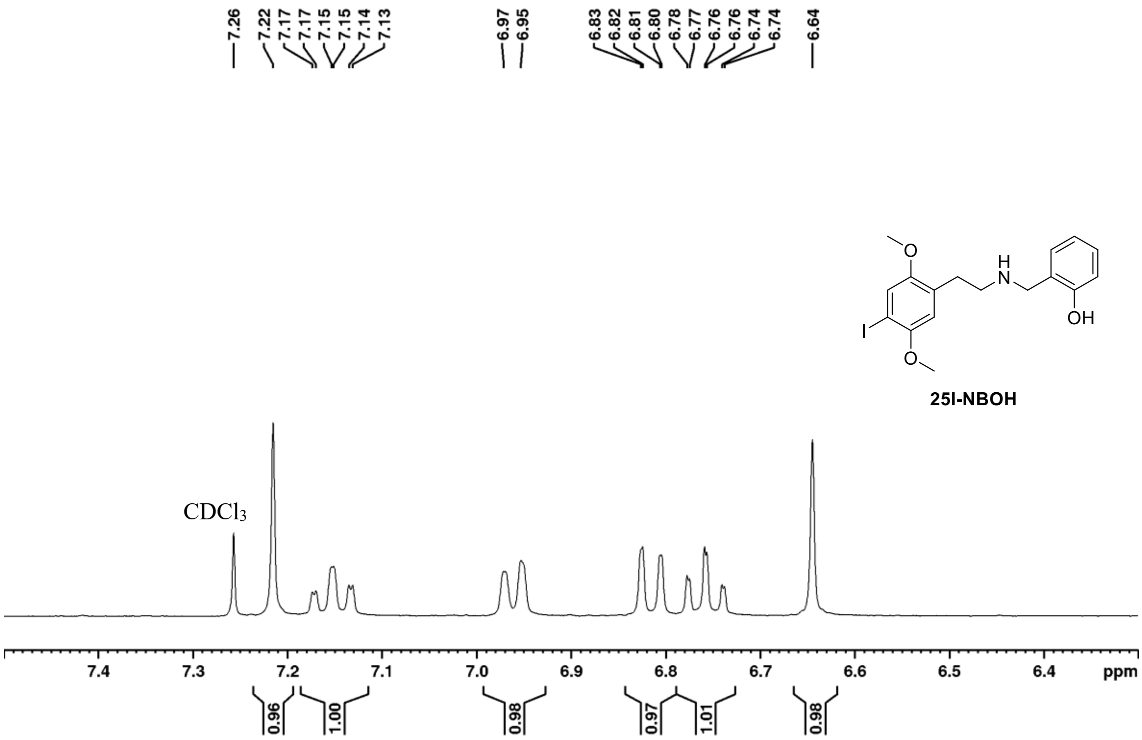

Figure S24. ^{1}H NMR (400 MHz, $CDCl_3$) of 25I-NBOH – Expanded.

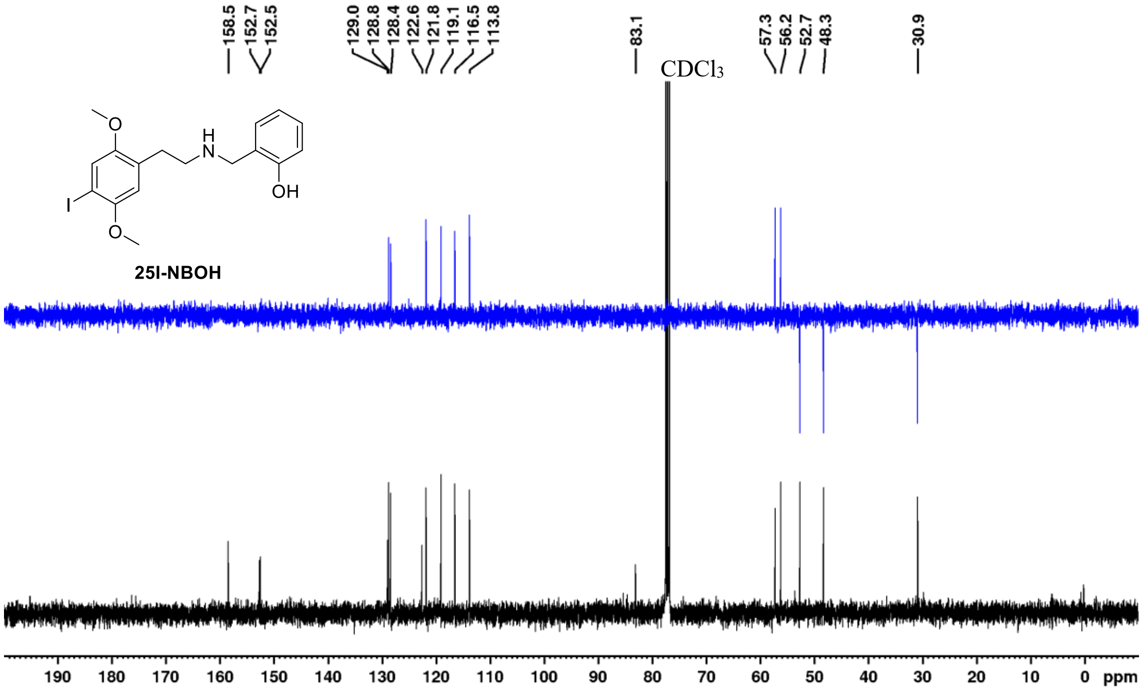

Figure S25. ^{13}C NMR (100 MHz, $CDCl_3$) of 25I-NBOH.

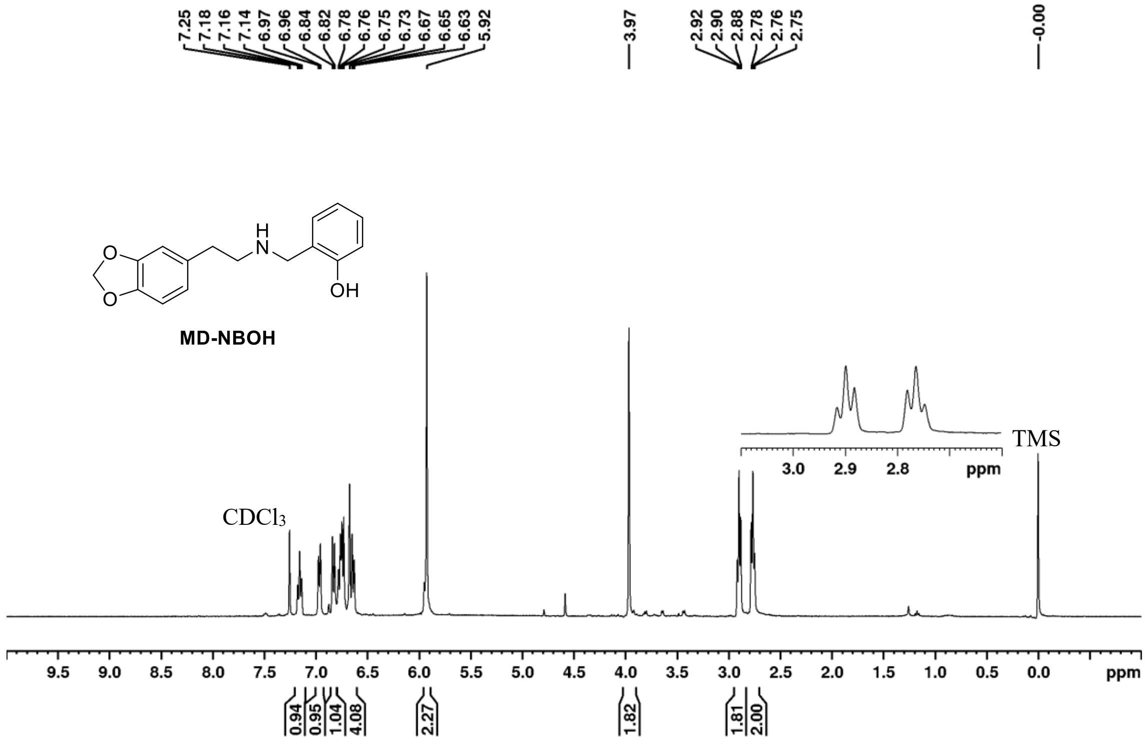

Figure S26. ^{1}H NMR (400 MHz, $CDCl_3$) of MD-NBOH.

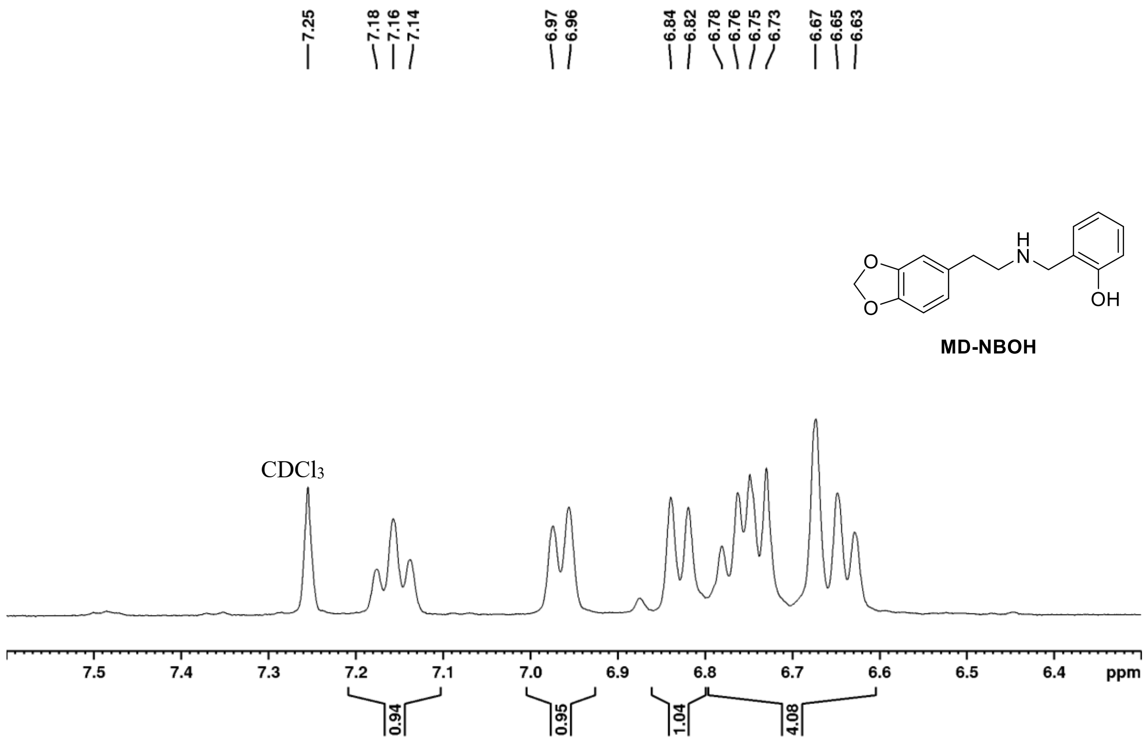

Figure S27. ^{1}H NMR (400 MHz, $CDCl_3$) of MD-NBOH – Expanded.

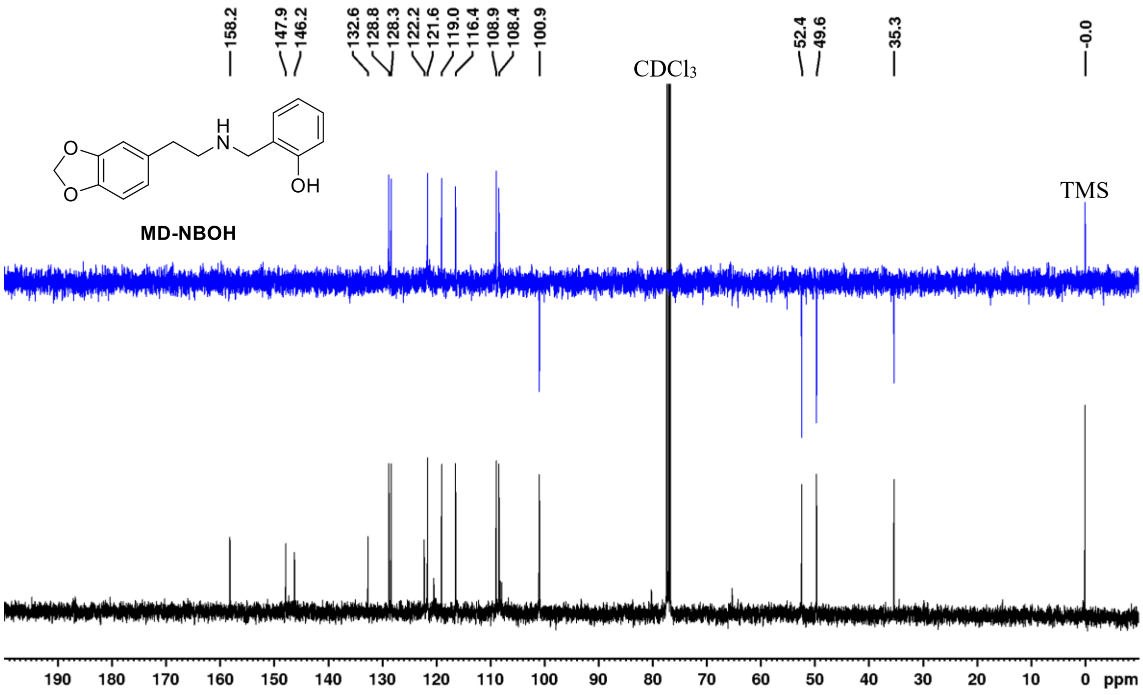

Figure S28. ^{13}C NMR (100 MHz, $CDCl_3$) of MD-NBOH.

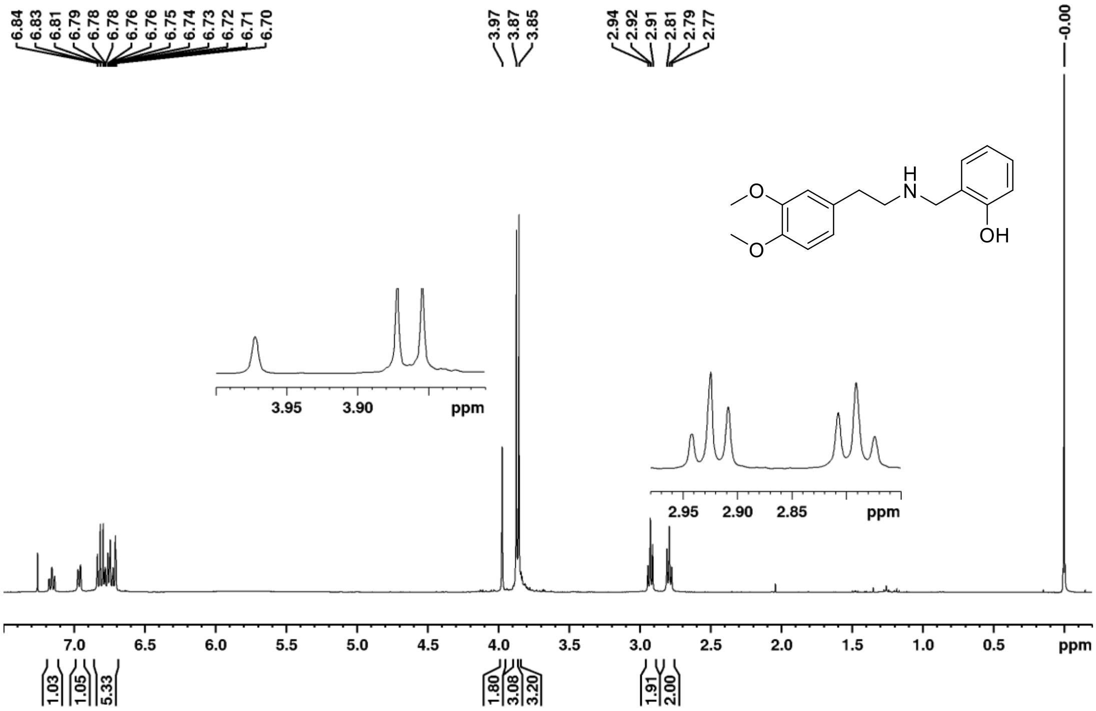

Figure S29. ^{1}H NMR (400 MHz, $CDCl_3$) of 34-NBOH

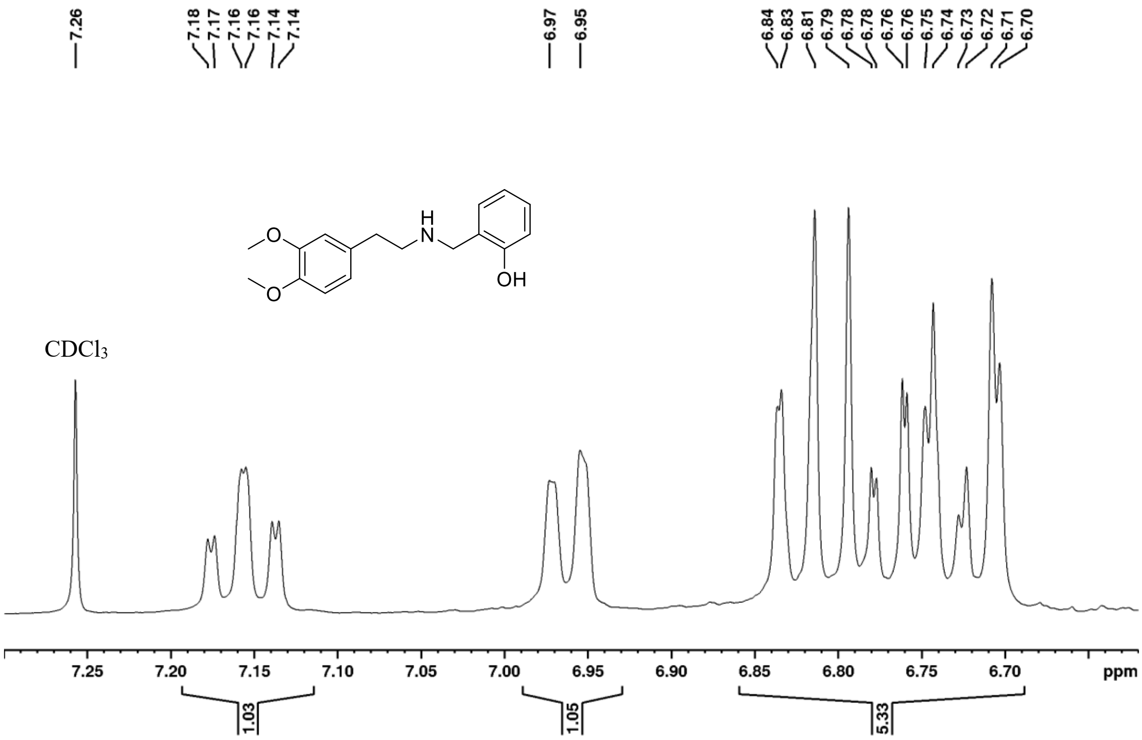

Figure S30. ^{1}H NMR (400 MHz, $CDCl_3$) of 34-NBOH – Expanded.

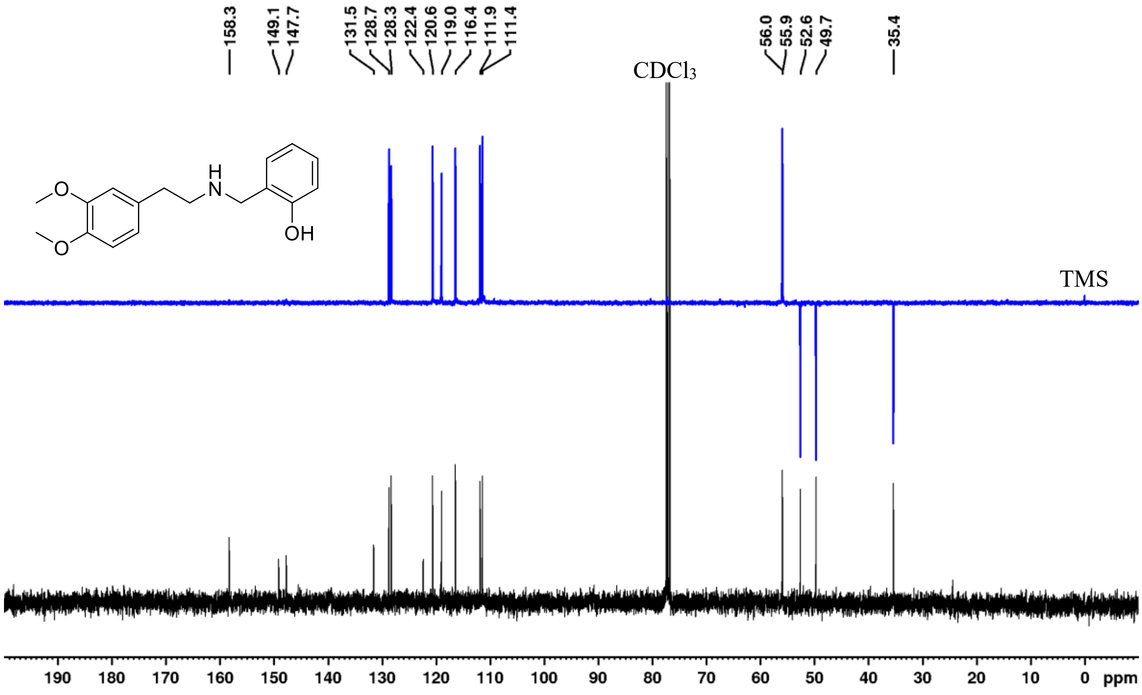

Figure S31. ^{13}C NMR (100 MHz, $CDCl_3$) of 34-NBOH.

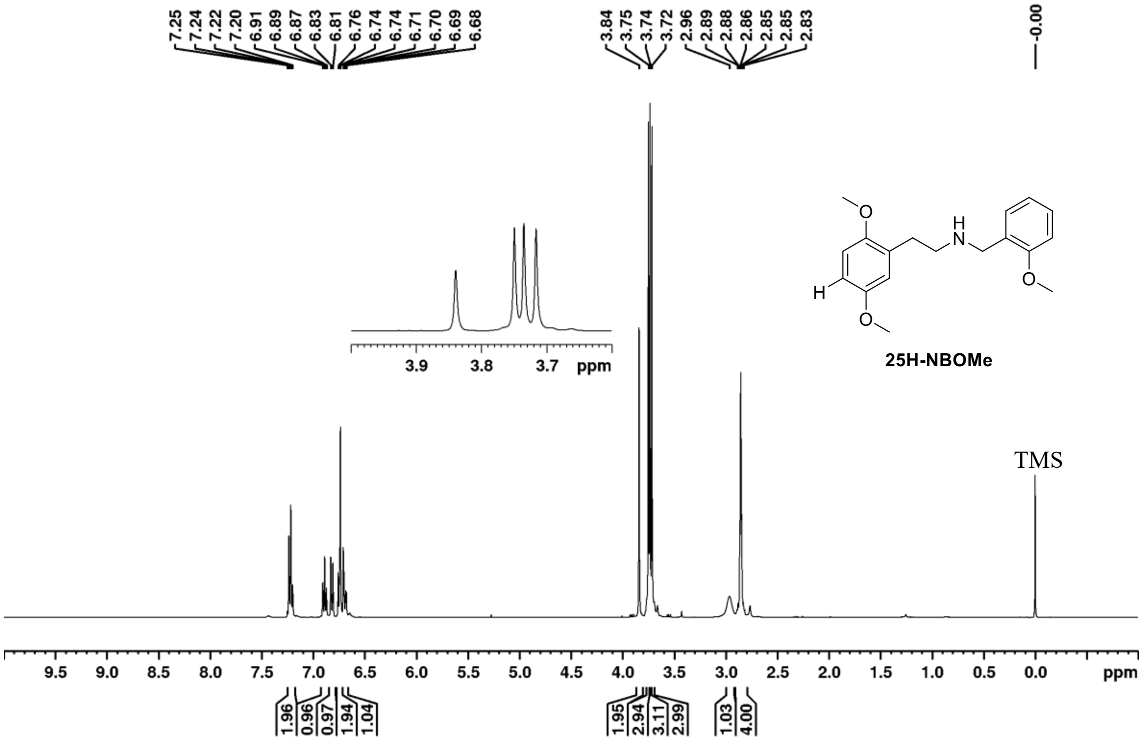

Figure S32. ^{1}H NMR (400 MHz, $CDCl_3$) of 25H-NBOMe.

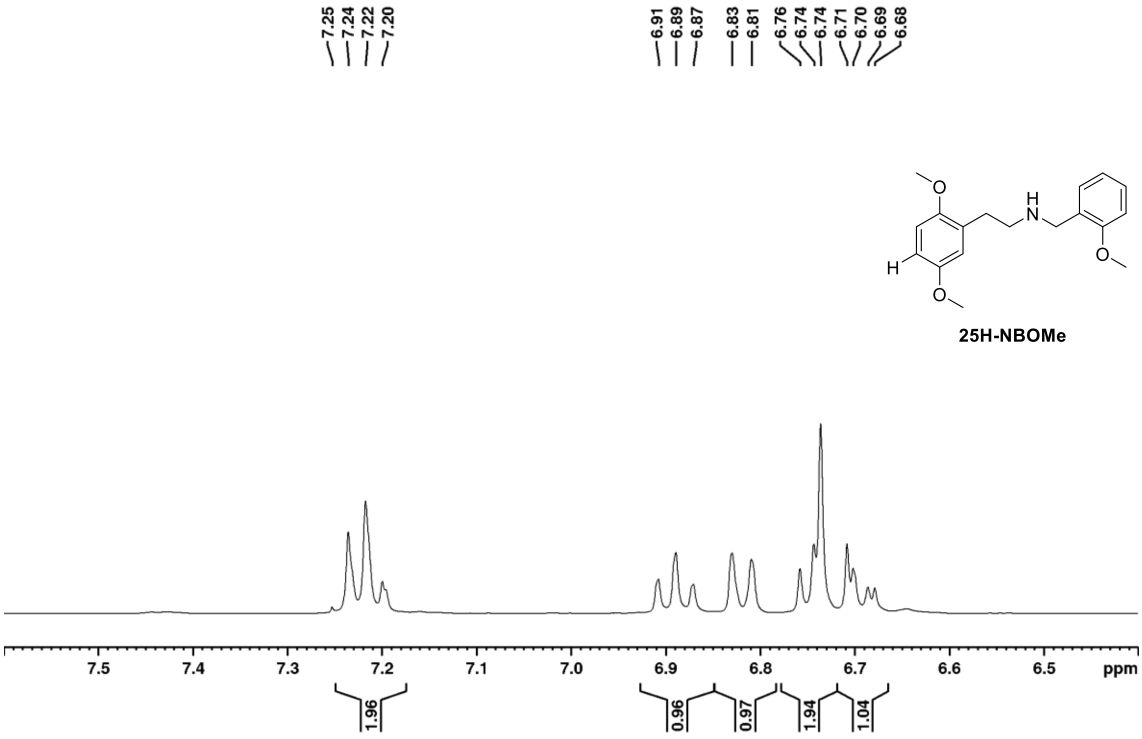

Figure S33. ^{1}H NMR (400 MHz, $CDCl_3$) of 25H-NBOMe – Expanded.

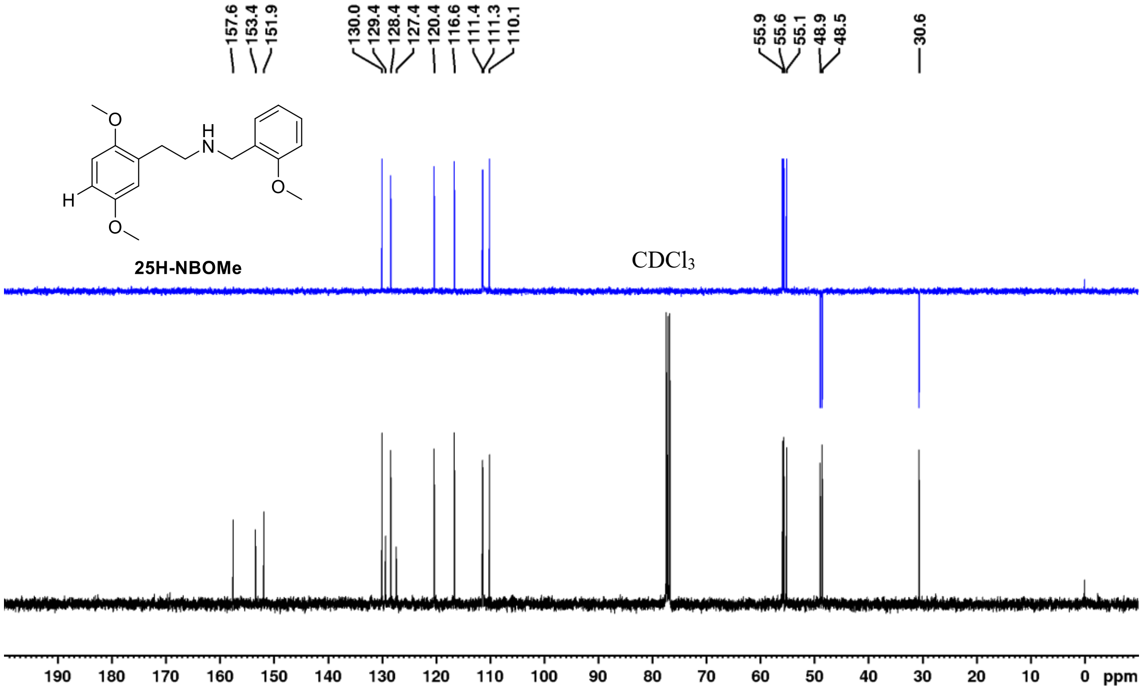

Figure S34. ^{13}C NMR (100 MHz, $CDCl_3$) of 25H-NBOMe.

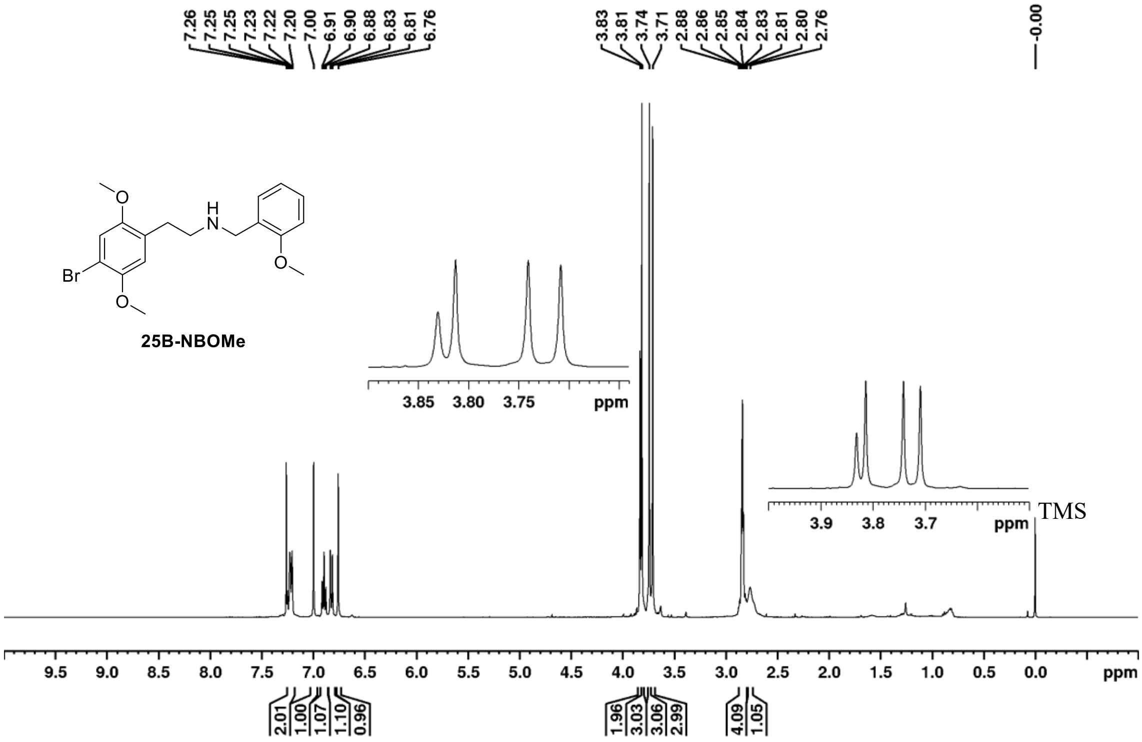

Figure S35. ^{1}H NMR (400 MHz, $CDCl_3$) of 25B-NBOMe**.**

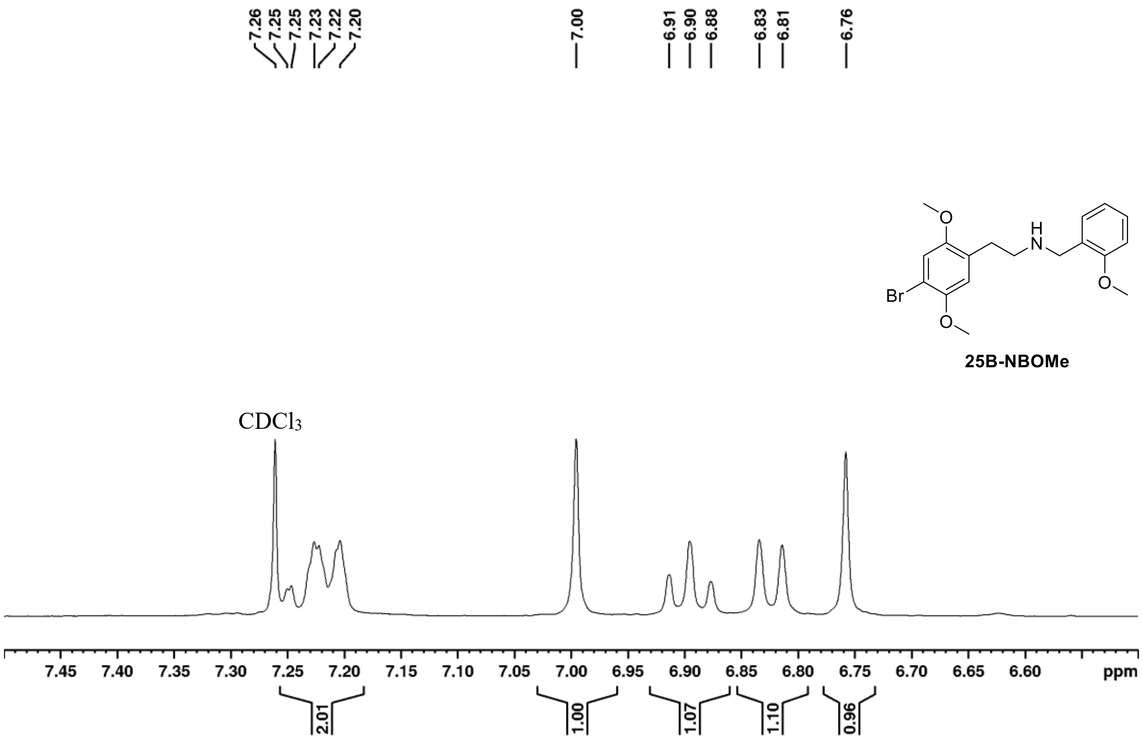

Figure S36. ^{1}H NMR (400 MHz, $CDCl_3$) of 25B-NBOMe – Expanded.

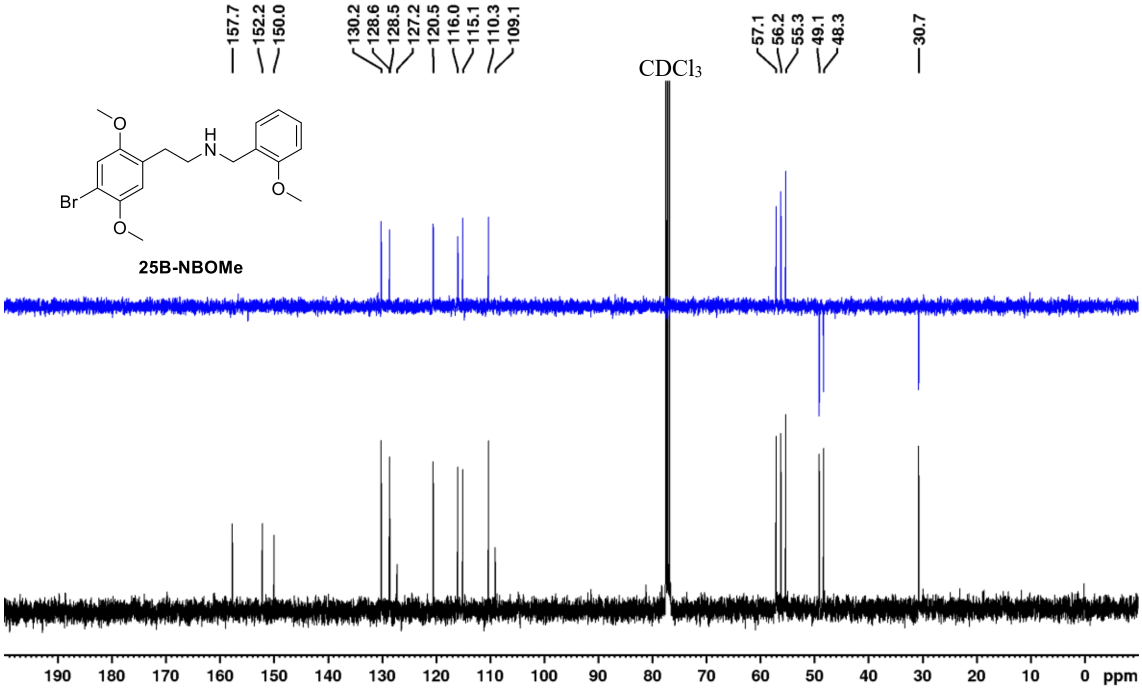

Figure S37: ^{13}C NMR (100 MHz, $CDCl_3$) of 25B-NBOMe.

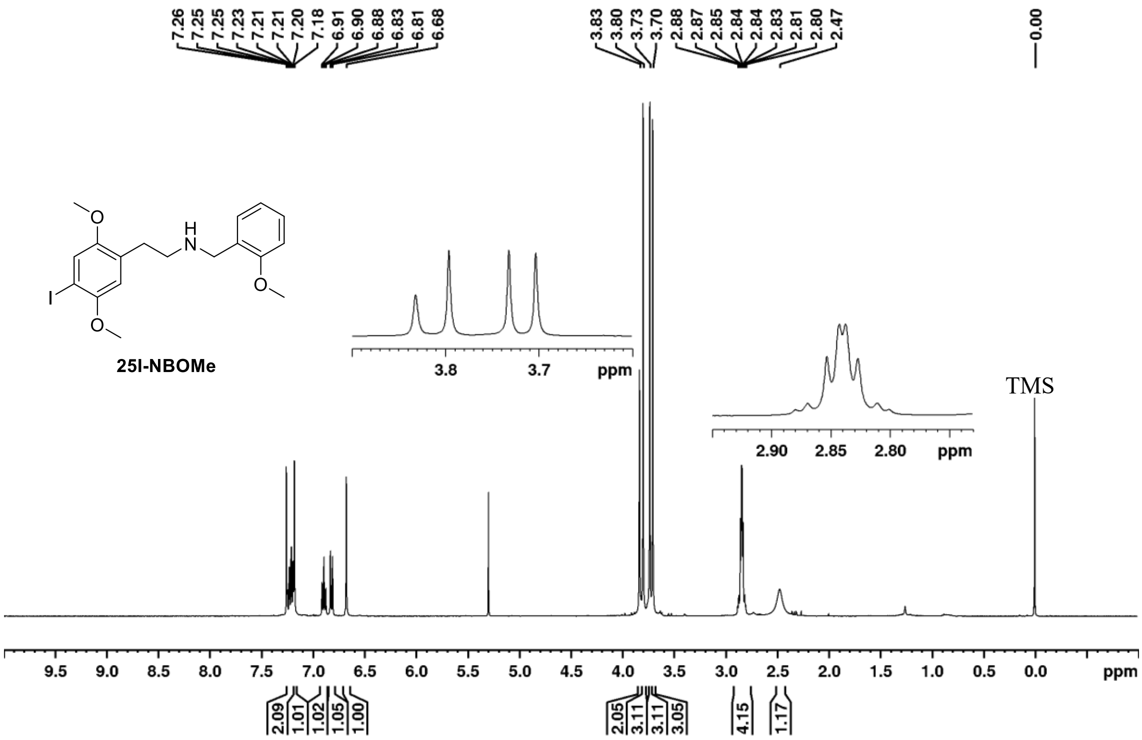

Figure S38. ^{1}H NMR (400 MHz, $CDCl_3$) of 25I-NBOMe**.**

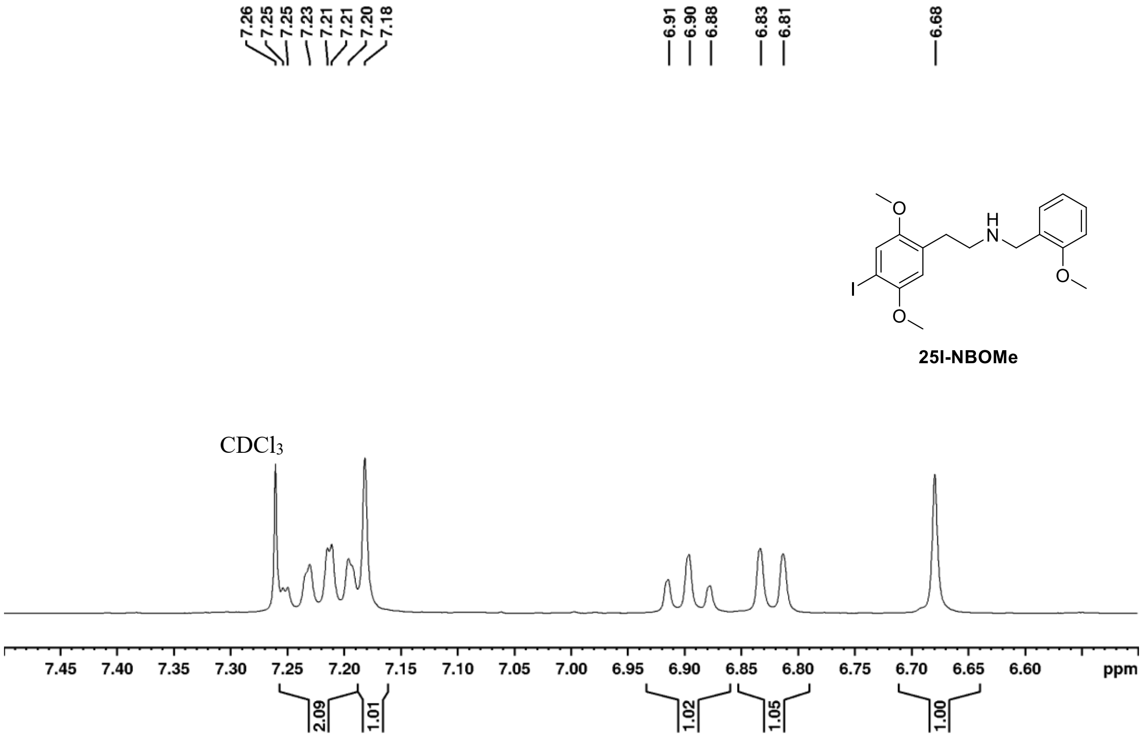

Figure S39. ^{1}H NMR (400 MHz, $CDCl_3$) of 25I-NBOMe – Expanded.

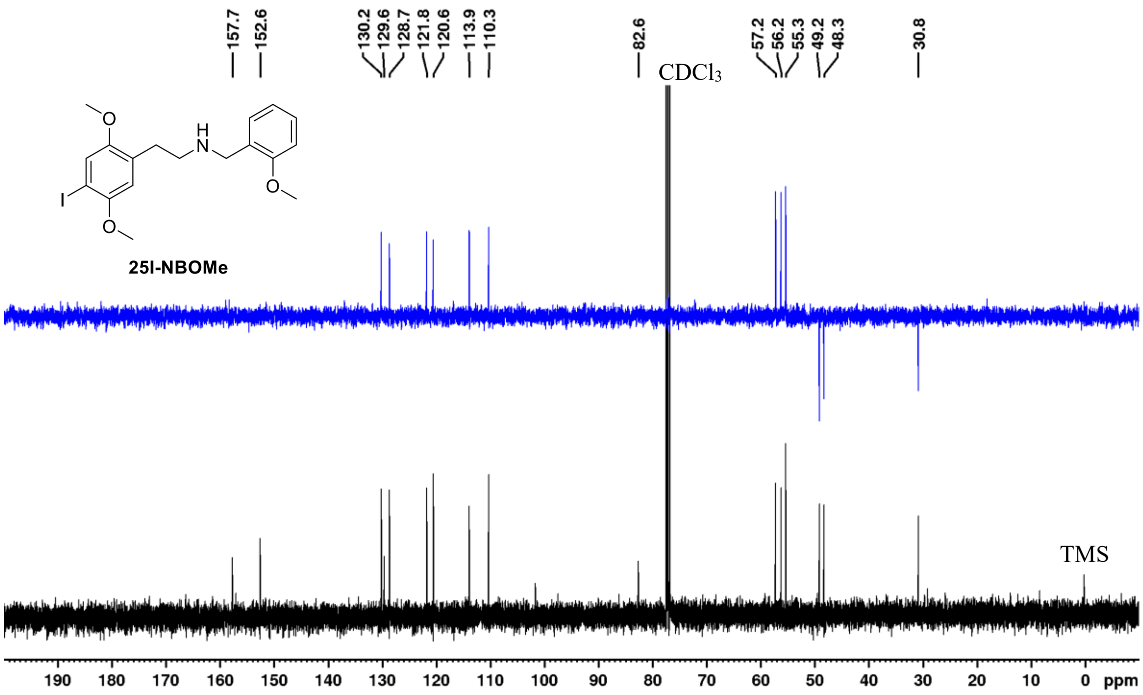

Figure S40. ^{13}C NMR (100 MHz, $CDCl_3$) of 25I-NBOMe.

REFERENCES

[1] W. Alves de Barros, M.P. Queiroz, L. da Silva Neto, G.M. Borges, F.T. Martins, Â. de Fátima, Synthesis of 25X-BOMes and 25X-NBOHs (X = H, I, Br) for pharmacological studies and as reference standards for forensic purposes, Tetrahedron Lett 66 (2021). https://doi.org/10.1016/j.tetlet.2020.152804.

[2] I. McConnell, E. Beckikh, P. Fitzgerald, A. P. Lowry, IMMUNOASSAY FOR COMPOUNDS OF THE NBOME FAMILY. United States Patent Application Publication, US 2015/0346226A1 (2005) 1-18

[3] X. Wu, C. Eriksson, A. Wohlfarth, J. Wallgren, R. Kronstrand, M. Josefsson, J. Dahlén, P. Konradsson, Synthesis and identification of metabolite biomarkers of 25C-NBOMe and 25I-NBOMe, Tetrahedron 73 (2017) 6393–6400. https://doi.org/10.1016/j.tet.2017.09.024.

[4] V.I. Vinogradova, T.I. Golodnyuk, M.S. Yunusov, G.A. Suleimanova, N. Tulyaganov, Syntheses based on β-phenylethylamines V. Synthesis and preliminary pharmacological screening of some phenylalkylamines and N-benzyltetrahydroisoquinolines, Chem Nat Compd 29 (1993) 654–657. https://doi.org/10.1007/BF00630218.

[5] M.M. Herth, S. Leth-Petersen, S. Lehel, M. Hansen, G.M. Knudsen, N. Gillings, J. Madsen, J.L. Kristensen, Accelerating preclinical PET-screening: reductive amination with [^{11}C]methoxybenzaldehydes, RSC Adv. 4 (2014) 21347–21350. https://doi.org/10.1039/C4RA02506G.
